# FGF2-primed 3D spheroids producing IL-8 promote therapeutic angiogenesis in murine hindlimb ischemia

**DOI:** 10.1038/s41536-021-00159-7

**Published:** 2021-08-18

**Authors:** Jungkyun Choi, Wooshik Choi, Yunji Joo, Haeun Chung, Dokyun Kim, Seung Ja Oh, Sang-Heon Kim

**Affiliations:** 1 Center for Biomaterials, Biomedical Research Institute, Korea Institute of Science and Technology (KIST), Seoul, Republic of Korea; 2 Division of Bio-Medical Science and Technology, KIST School, Korea University of Science and Technology, Seoul, Republic of Korea

**Keywords:** Cell delivery, Peripheral vascular disease, Mesenchymal stem cells, Angiogenesis, Regenerative medicine

## Abstract

Peripheral artery disease is a progressive, devastating disease that leads to critical limb ischemia (CLI). Therapeutic angiogenesis using stem cell therapy has emerged as a promising approach for its treatment; however, adapting cell-based therapy has been limited by poor cell survival and low treatment efficiency. To overcome unmet clinical needs, we developed a fibroblast growth factor 2 (FGF2)-immobilized matrix that enabled control of cell adhesion to the surface and exerted a priming effect on the cell. Human adipose-derived stem cells (hASCs) grown in this matrix formed a functionally enhanced cells spheroid (FECS-Ad) that secreted various angiogenic factors including interleukin-8 (IL-8). We demonstrated that IL-8 was upregulated by the FGF2-mediated priming effect during FECS-Ad formation. Immobilized FGF2 substrate induced stronger IL-8 expression than soluble FGF2 ligands, presumably through the FGFR1/JNK/NF-κB signaling cascade. In IL-8-silenced FECS-Ad, vascular endothelial growth factor (VEGF) expression was decreased and angiogenic potential was reduced. Intramuscular injection of FECS-Ad promoted angiogenesis and muscle regeneration in mouse ischemic tissue, while IL-8 silencing in FECS-Ad inhibited these effects. Taken together, our data demonstrate that IL-8 contributes to therapeutic angiogenesis and suggest that FECS-Ad generated using the MBP-FGF2 matrix might provide a reliable platform for developing therapeutic agents to treat CLI.

## Introduction

**Fig. 1:**
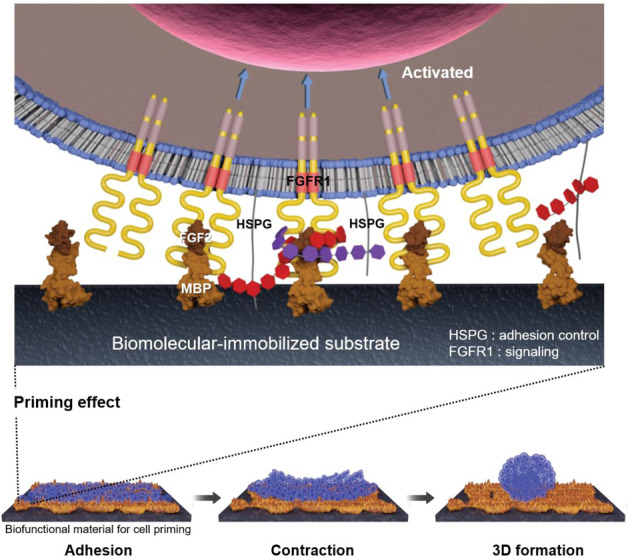
Schematic illustration of MBP-FGF2 action on 3D formation and priming of hASCs. The MBP-FGF2 protein was immobilized on polystyrene plates. This biologically modified substrate could trigger an intracellular signaling pathway through binding with the membrane receptor, FGFR1. It also contributed to forming cellular spheroids by adhering to HSPG to control cell-matrix adhesion strength.

Critical limb ischemia (CLI) is the most severe clinical manifestation of peripheral arterial disease (PAD), which is caused by atherosclerotic changes in arteries supplying blood to the lower extremities. Prolonged ischemic conditions frequently lead to disability and amputation of the affected limb, which greatly reduces quality of life^1^. The current available clinical treatments for this disease are endovascular or surgical revascularization, which are limited by morbidity and mortality complications^2^. Therefore, there is an urgent need for novel therapeutic strategies for patients with CLI. The term “therapeutic angiogenesis” refers to biological intervention to stimulate the creation of new blood vessels in ischemic organs or tissues by administering angiogenic factors^3^. Several factors tightly regulate vascularization and angiogenic progress. Well-characterized angiogenic factors include vascular endothelial growth factor (VEGF), angiopoietins, fibroblast growth factor (FGF), hepatocyte growth factor (HGF), and platelet-derived growth factor (PDGF), although dozens of other proteins contribute to the different stages of blood vessel formation. For example, VEGF plays a role in increasing the number of capillaries, and angiopoietins contribute to blood vessel stability and maturation^4^. Under normal physiological conditions, most of these secretory factors are tethered to components of the extracellular matrix (ECM), including heparan sulfate proteoglycans (HSPGs) and fibronectin. During tissue injury, mechanical or biological stimuli such as low oxygen concentration (hypoxia) induce the cleavage of ECM by hydrolytic enzymes to release proangiogenic factors toward the damaged tissue^5^. Strategies for therapeutic angiogenesis have been widely investigated for application in diverse human diseases. These include nucleotide or protein delivery and cell transplantation. However, treatments for therapeutic angiogenesis have not yet been approved by the U.S. Food and Drug Administration^6^. Recent advances in stem cell therapy have recognized the therapeutic potential of mesenchymal stem cells (MSCs) for ischemic diseases, such as CLI and myocardial infarction^6^. In particular, human adipose-derived stem cells (hASCs) have gained particular attention since they are readily available, abundantly supplied among MSCs, and also retain immunoprivileged properties and the ability to differentiate multiple cell lineages^7,8^. They also reportedly promote angiogenesis by secreting various angiogenic factors. Despite the advantages of these cell sources, unsolved limitations regarding their clinical application remain. These mainly include insufficient therapeutic efficacy and poor cell survival rates due to limited well-organized vascular tissue at the transplanted site. Additionally, cells administered into the ischemic region are exposed to hypoxia, causing cell apoptosis^9^. Several methods have been developed to overcome these therapeutic angiogenesis limitations, including: (1) transplanting cells with additional cell-supporting components, (2) genetically modifying cells to enhance functionality and survival, and (3) three-dimensional (3D) cell clustering. However, current methods utilizing transduced cells or cells combined with growth factors are extremely limited as they can potentially show immunogenicity and oncogenicity^10^. On the other hand, 3D cell clusters, especially in scaffold-free platforms, reportedly exert therapeutic effects in ischemic diseases by inducing cell growth and survival without such safety issues. Among several 3D clustering platforms, physically modified surfaces, such as low attachment and patterned surfaces, were utilized for cell spheroid formation as they are relatively easy to control and suitable for the formation of uniform 3D structures without shear stress^11^. We designed a biologically modified surface to realize a functionally enhanced cell spheroid (FECS) for high-quality cell therapeutics. As shown in Fig. 1, a biologically modified surface plays multiple roles in enhancing cell function through cell membrane receptor signaling and forms cellular spheroids through control of the cell-matrix adhesion strength. We previously developed a novel surface with biological functionality: the maltose-binding protein-fused basic fibroblast growth factor (MBP-FGF2)-coated surface^12^. MBP is a periplasmic receptor for maltose transport, originally found in Gram-negative bacteria. MBP-fusion protein expression systems are commercially available and have been used for purification of recombinant proteins^13^. Since MBP has considerable hydrophobic residues on its surface, the protein enables MBP-fused bioactive molecules to coat hydrophobic polystyrene (PS) plates. We demonstrated that various MBP-fused proteins, including MBP-FGF2, could be coated onto a PS surface as a monolayer^14,15^. We previously reported that hASCs could uniformly form 3D spheroids, which we named FECS-Ad, on this surface^16^. FGF2 in the MBP-FGF2-coated surface mediated coupling with the cell surface in a heparan sulfate proteoglycan (HSPG)-dependent manner, which led to reduced cell-to-matrix adhesion^17^. The biophysical balance between cell-to-cell contact and cell-to-surface adhesion force may be involved in the generation of 3D spheroids by inducing aggregation of cells^18^. Cells in 3D culture, including spheroids, secrete various growth factors and cytokines, but the mechanisms by which this occurs remain to be elucidated. FECS-Ad secreted various angiogenic factors, including VEGF, FGF2, HGF, and IL-8 in vitro, and promoted angiogenesis in a mouse ischemic model^16^. As shown in Fig. 1, the principle of enhanced cell function in FECS-Ad was hypothesized to be due to 3D formation, hypoxia, and FGF2-mediated cell signaling. We previously demonstrated that VEGF expression was regulated by hypoxia induced during 3D clustering^16^, and other studies have reported that HGF was induced by 3D formation-mediated stimuli^19^. It has been reported that IL-8, an emerging regulator of angiogenesis, might be regulated via growth factor signaling, such as the FGF2 signaling pathway^20,21^. FGF2 is a well-characterized growth factor involved in various cellular responses, including embryonic development, cell growth, and tissue repair. Interaction of FGF2 with its cellular receptors, primarily FGFR1, activates a variety of signaling pathways such as PI3K, MAPK, and STAT^22^. Further, FGF2 is known to bind to HSPGs, surface molecules that play important roles in cell motility and organization. Binding of FGF2 to HSPGs reportedly boosted the signaling cascade by stabilizing the FGF2-FGFR1 complex^23^. In this study, we investigated the angiogenic potential of FECS-Ad in a murine hindlimb ischemia model, and explored IL-8 function in this process. Our study findings suggested that FECS-Ad and MBP-FGF2 may provide valuable inspiration for developing stem cell therapeutics based on bio-functional materials for treatment of CLI.

## Results

### MBP-FGF2 matrix induces IL-8 expression during 3D spheroid formation

**Fig. 2:**
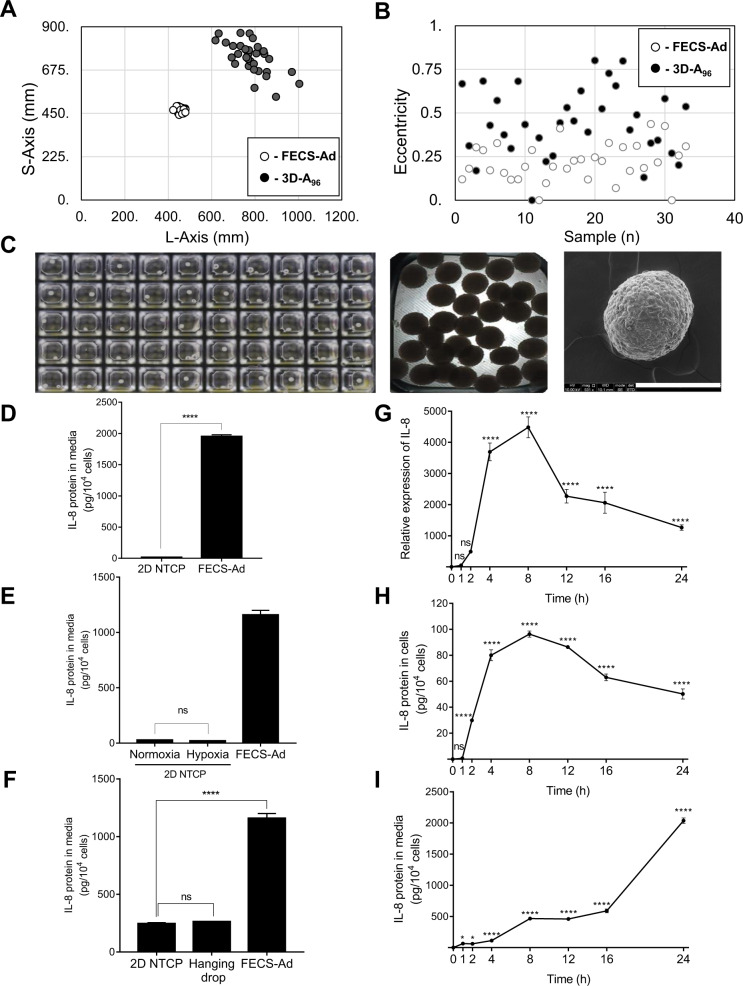
MBP-FGF2-mediated priming induced IL-8 expression during 3D formation. **A**–**C** Characterization of 3D-As. **A** Size ranges of FECS-Ad and 3D-A_96_. *n* = 33 per group. **B** Shape distribution of 3D-As. *n* = 33 per group. **C** Digital optical, phase contrast, and SEM images of FECS-Ad formed in a 384-well microplate, Scale bar, 200 μm. **D**–**F** hASCs were cultured on MBP-FGF2 surface, under hypoxia, or using the hanging drop method for 24 h. Conditioned medium were prepared followed by ELISA. **D** IL-8 protein expression in FECS-Ad. ****p < 0.0001 (unpaired Student’s *t* test), *n* = 6 per group. **E** Effect of hypoxic condition on IL-8 protein. ns not significant (one-way ANOVA), *n* = 6 per group. **F** IL-8 production in 3D spheroids generated by the hanging drop. ****p < 0.0001 (one-way ANOVA), *n* = 6 per group. **G**–**I** hASCs were cultured on MBP-FGF2 surface in a monolayer manner for appropriate time points. Total RNA, protein, and conditioned medium were prepared and analyzed by RT-qPCR and ELISA, respectively. **G** IL-8 RNA expression on MBP-FGF2 matrix. Values were normalized to GAPDH. ****p < 0.0001 (one-way ANOVA), *n* = 3 per group. **H** IL-8 protein expression in hASCs attached to MBP-FGF2 surface. ****p < 0.0001 (one-way ANOVA), *n* = 3 per group. **I** IL-8 protein secretion on MBP-FGF2 surface. ****p < 0.0001 (one-way ANOVA), *n* = 3 per group. All data are presented as mean ± SD.

To generate 3D spheroids, hASCs were seeded at a density of 1 × 10^5^ cells/cm^2^ in microplates coated with MBP-FGF2. Cells began to aggregate 4 h after seeding and gradually became compact to form 3D spherical clusters (3D-A) in each well within 24 h (Supplementary Video 1 and Supplementary Fig. 1A). More uniform and spherical 3D-As formed in the 96- and 384-well MBP-FGF2-coated microplates than in other plates (data not shown). Notably, when we compared the 384-well (FECS-Ad) and 96-well (3D-A_96_) plates, 3D-A_96_ size was broadly distributed from 470 to 900 μm, whereas the average FECS-Ad size was 470 μm with a standard deviation of <5% (Fig. 2A). Furthermore, the eccentricity (a measure of deviation from spherical shape) of FECS-Ad was <0.5, whereas that of 40% of the spheres in the 3D-A_96_ wells was >0.5 (Fig. 2B). As shown in Fig. 2C, only one 3D-A formation was observed in each of the wells of the MBP-FGF2-coated microplate. FECS-Ad spheres exhibited uniform morphology with respect to size and nearly spherical shape, observed using phase-contrast microscopy and scanning electron microscopy (SEM). These results indicated that FECS-Ad was more suitable for forming 3D-As of uniform size and shape, and could therefore comply with standardization requirements of manufacturing processes. During 3D spheroid formation, hypoxic conditions and stimuli followed by 3D construction within the spheroid reportedly contribute to the induction of various secretory factors. For example, it was reported that 3D formation of the mesenchymal stromal progenitors induced an increased secretion of extracellular vesicles (EVs)^24,25^. The concentration of EVs was observed to be much higher in FECS-Ad-conditioned medium (4.36 × 10^9^ ± 1.80 × 10^8^ particles/ml) compared to that from monolayer hASCs (1.30 × 10^9^ ± 9.70 × 10^7^ particles/ml). The size of EVs was not significantly different between the two groups (Supplementary Fig. 1B). In addition, we found that hypoxia regulated expression of VEGF, and 3D formation-mediated stimuli was the main factor responsible for the increased HGF expression (Supplementary Fig. 1C, D)^16^. The novel MBP-FGF2-immobilized substrate generated uniform 3D spheroids, which conferred not only a hypoxic environment and aggregating force, but also activated the FGF2-mediated signaling pathway. To investigate the possible contribution of FGF2-mediated priming to cells, IL-8 expression was analyzed during FECS-Ad formation. As shown in Fig. 2D, IL-8 secretion was significantly increased in FECS-Ad compared to that in non-tissue culture plate (NTCP)-attached cells. To examine the direct influence of hypoxia on IL-8 expression in FECS-Ad, hASCs were seeded on NTCP and incubated in a hypoxic (1% O_2_) environment for 24 h, and IL-8 protein secretion was analyzed. IL-8 secretion between cells exposed to normoxic (20% O_2_) and hypoxic environments did not differ significantly, suggesting that hypoxia does not participate in the expression of IL-8 (Fig. 2E). The effect of 3D formation stimuli on IL-8 secretion was also investigated. In 3D spheroids formed using the hanging drop method^26^, IL-8 expression remained unchanged compared to hASCs, but was drastically increased in spheroids generated using the MBP-FGF2-immobilized substrate (Fig. 2F). Next, we tested the effect of cell priming by MBP-FGF2 on IL-8 expression in hASCs. To eliminate possible effects that may result from hypoxia or 3D formation during culturing on the MBP-FGF2 matrix, hASCs were cultured at a lower cell density (1.5 × 10^4^ cells/cm^2^) on the MBP-FGF2 surface to maintain a monolayer state. RNA and protein levels of IL-8 in hASCs gradually increased, reached a peak at 8 h, and then steadily decreased during the 24 h period after attachment to the MBP-FGF2 substrate (Fig. 2G, H). IL-8 protein secretion continuously increased over 24 h, accumulating in the media (Fig. 2I). These results indicated that increased IL-8 expression during the formation of FECS-Ad was mainly attributed to the MBP-FGF2 matrix, presumably by triggering an intracellular signaling pathway.

### MBP-FGF2 regulates IL-8 expression via FGFR1 signaling

**Fig. 3:**
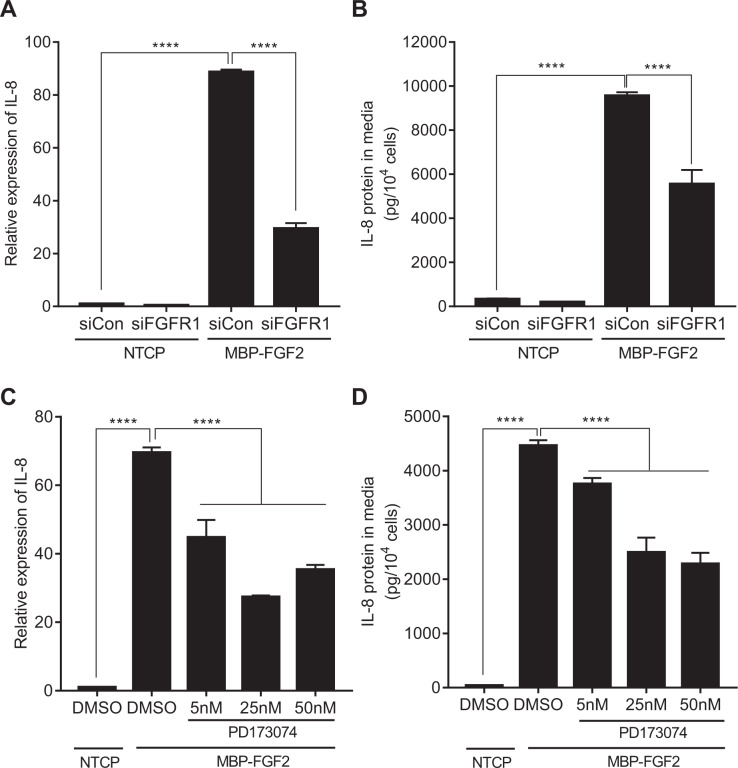
Roles of FGFR1 on the MBP-FGF2-mediated IL-8 expression. hASCs were treated with FGFR1 siRNA or PD173074, an FGFR1 inhibitor, and cultured on NTCP or MBP-FGF2 surface for 24 h. Total RNA and conditioned medium were prepared and analyzed by RT-qPCR and ELISA, respectively. **A** Effect of FGFR1 knockdown on IL-8 RNA. **B** Effect of FGFR1 knockdown on IL-8 protein. **C** Effect of PD173074 on IL-8 RNA. **D** Effect of PD173074 on IL-8 protein. For all data, ****p < 0.0001 (one-way ANOVA), *n* = 3 per group. Values were normalized to GAPDH for RT-qPCR analysis. All data are presented as mean ± SD.

It was hypothesized that the FGF2 domain in MBP-FGF2 might be able to bind to its canonical receptor, FGFR1, in the membrane of hASCs. To test the effect of FGFR1 on the MBP-FGF2 matrix-mediated increase in IL-8 expression, hASCs were transfected with siRNA against FGFR1, followed by examination of IL-8 RNA and protein levels in cells adhered to the MBP-FGF2 surface. Expression of FGFR1 was highly reduced by siRNA transfection (Supplementary Fig. 2A, B). The RNA level of IL-8 was greatly increased when cells were attached to the MBP-FGF2 substrate, and was significantly reduced by FGFR1 knockdown (Fig. 3A). IL-8 protein expression exhibited a similar pattern; it was decreased to ~57% compared to the control when transfected with FGFR1 siRNA (Fig. 3B). Next, the effect of PD173074, an inhibitor of FGFR1, on IL-8 expression was tested. Attachment to the MBP-FGF2 surface increased RNA and protein levels of IL-8, while the presence of PD173074 decreased IL-8 expression in a dose-dependent manner (Fig. 3C, D). To test the possible involvement of HSPGs, exotosin-1, a protein that plays an essential role in the synthesis of HSPGs, was silenced and expression of IL-8 was investigated. However, exotosin-1 silencing in hASCs did not significantly affect IL-8 expression, suggesting that HSPGs play a negligible role in the regulation of IL-8 by MBP-FGF2 (Supplementary Figs. 3A–C). These results suggested that the MBP-FGF2 matrix might control IL-8 expression through FGFR1 in hASCs.

### MBP-FGF2 regulates JNK-NF-κB to control IL-8 expression

**Fig. 4:**
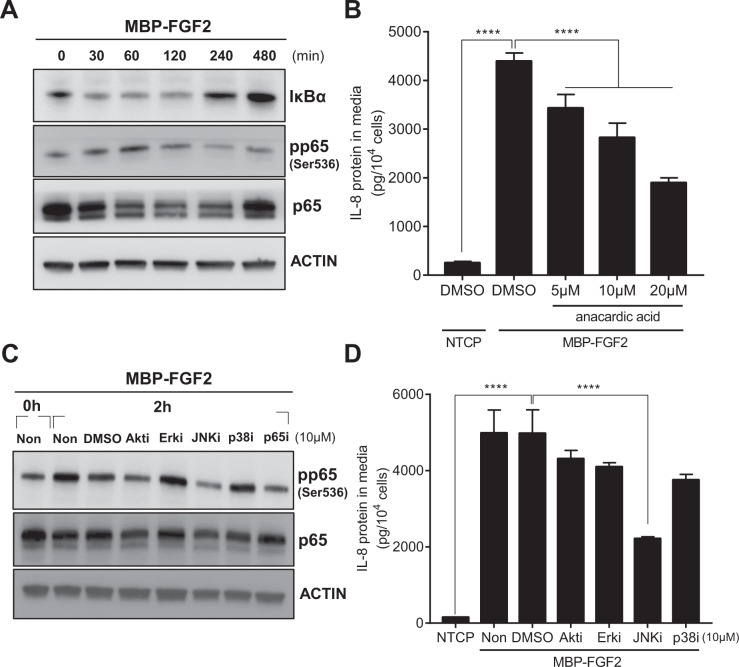
Regulation of IL-8 expression of MBP-FGF2 through JNK-NF-κB cascade. hASCs were cultured on MBP-FGF2 surface in the presence of various inhibitors for downstream molecules. Total proteins and conditioned medium were prepared and analyzed by western blot and ELISA, respectively. **A** Time-dependent activation of NF-κB signaling pathway of hASC on the MBP-FGF2 surface. **B** Effect of anacardic acid on IL-8 protein expression. ****p < 0.0001 (one-way ANOVA), *n* = 4 per group. **C** Effect of various chemical inhibitors on the MBP-FGF2-mediated increase of phosphorylated p65. **D** Effect of chemical inhibitors on the MBP-FGF2-mediated regulation of IL-8 expression. ****p < 0.0001 (one-way ANOVA), *n* = 4 per group. For western blot analysis, β-actin was used as a loading control. All data are presented as mean ± SD.

The contribution of downstream FGFR1 signaling to the production of MBP-FGF2 matrix-induced IL-8 was investigated. Among various factors, NF-κB is an inducible dimeric transcription factor known to be involved in the expression of IL-8^21^. To test the possible involvement of NF-κB in IL-8 secretion, hASCs were cultured on the MBP-FGF2 surface, and NF-κB activity was investigated through phosphorylation of the NF-κB p65 subunit. NF-κB activity gradually increased, peaked at 1 h, and steadily decreased before returning to the basal level at 8 h. Concurrently, expression of IκBα, a negative regulator of NF-κB activity, was reduced by attachment to MBP-FGF2, and was progressively upregulated to the basal condition (Fig. 4A). Next, the effect of anacardic acid, an inhibitor of NF-κB, on IL-8 expression was tested. As shown in Fig. 4B, attachment of hASCs to the MBP-FGF2 matrix increased IL-8 expression, and treatment of cells with the NF-κB inhibitor dose-dependently lowered the IL-8 protein level. Therefore, NF-κB appeared to act as a downstream regulator of MBP-FGF2-mediated production of IL-8 in hASCs. FGF2/FGFR1 signaling is known to utilize downstream effectors such as PI3K-Akt and mitogen-activated protein kinases p38, Erk, and JNK to induce various cellular responses. The involvement of downstream FGF2/FGFR1 signaling effectors in the induction of NF-κB activity was examined by treating hASCs with inhibitors of PI3K (LY294002), Erk (U0126), JNK (SP600125), p38 (SB203580), and NF-κB (anacardic acid). Again, adherence to MBP-FGF2 increased phosphorylation of the p65 subunit. Among inhibitors, only the JNK inhibitor was able to reduce p65 phosphorylation to a similar extent as the NF-κB inhibitor (Fig. 4C). Consistent with these data, treatment with the JNK inhibitor significantly reduced IL-8 expression mediated by MBP-FGF2 (Fig. 4D). We further investigated the activation of FGF2-mediated JNK-NF-κB cascade at different time points during FECS-Ad formation. The activity of JNK and NF-κB peaked at 2 h, and steadily decreased before returning to the basal level at 24 h, suggesting that the activity of FGF2 signaling might correlate with the degree of hASC attachment to the MBP-FGF2 matrix (Supplementary Fig. 2D). Taken together, these results indicated that the JNK-NF-κB cascade might act as a downstream signal of the FGF2-FGFR1 pathway to induce IL-8 expression.

### MBP-FGF2 matrix boosted the JNK-NF-κB-IL-8 signaling cascade

**Fig. 5:**
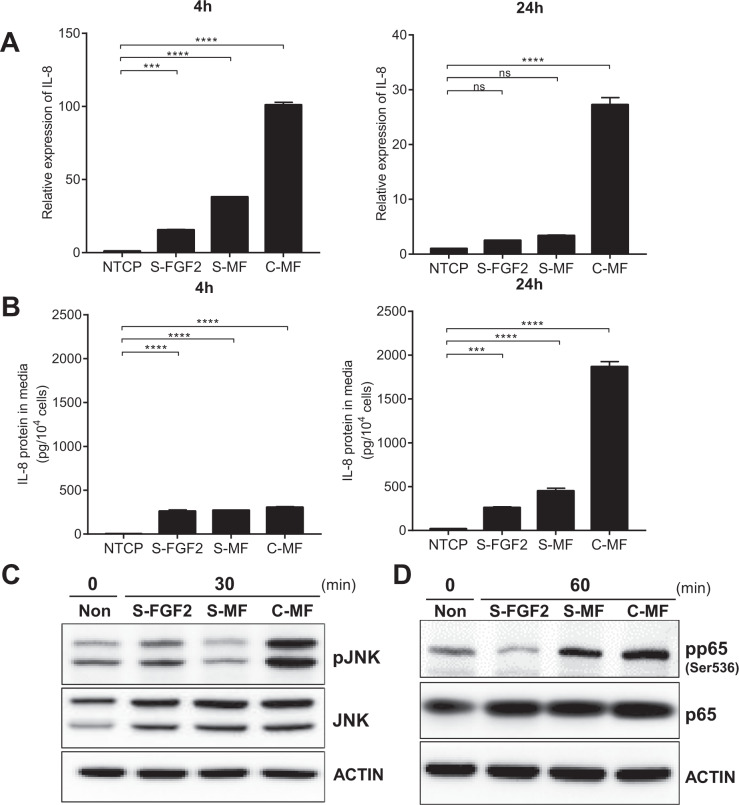
Enhanced JNK-NF-κB signaling and IL-8 expression on MBP-FGF2 matrix. hASCs were cultured on MBP-FGF2 surface, or treated with soluble form of FGF2, or MBP-FGF2 proteins. Total RNAs, proteins, and conditioned medium were prepared and analyzed by RT-qPCR, western blot, and ELISA, respectively. **A** Effect of various FGF2 ligands on IL-8 RNA. Values were normalized to GAPDH. ns not significant, ***p < 0.001, ****p < 0.0001 (one-way ANOVA), *n* = 3 per group. **B** Effect on IL-8 protein. ***p < 0.001, ****p < 0.0001 (one-way ANOVA), *n* = 3 per group. **C** Effect on JNK phosphorylation. **D** Effect on p65 phosphorylation. For western blot analysis, β-actin was used as a loading control. All data are presented as mean ± SD.

The immobilization of signaling molecules, such as growth factors, reportedly increases downstream signaling^27^. Therefore, we investigated IL-8 expression in hASCs exposed to various FGF2 ligands. For soluble ligands, recombinant FGF2 and MBP-FGF2 proteins were used. IL-8 expression reached a plateau at a concentration of 1000 ng/mL when cells were exposed to the three FGF2 ligands: MBP-FGF2 matrix (C-MF), soluble FGF2 (S-FGF2), and soluble MBP-FGF2 (S-MF) proteins. Therefore, 1000 ng/mL of C-MF, S-FGF2, and S-MF were used for further studies (Supplementary Figs. 4A–D). The RNA level of IL-8 was significantly upregulated when cells were exposed to each FGF2 ligand, and was much higher in the C-MF group than in the S-FGF2 and S-MF groups (Fig. 5A). IL-8 protein expression was also increased in hASCs after exposure to FGF2 ligands. Expression of IL-8 protein did not differ significantly among the S-FGF2, S-MF, and C-MF groups after 4 h; however, the protein level was drastically elevated in the C-MF group compared to the S-FGF2 and S-MF groups after 24 h (Fig. 5B).

Next, the effect of S-FGF2, S-MF, and C-MF on JNK-NF-κB activity was investigated. Various FGF2 ligands were exposed to hASCs for 30 and 60 min, which is when the phosphorylation of JNK and p65 peaked after attachment to the MBP-FGF2 matrix, respectively (Fig. 4A and Supplementary Fig. 2C). As shown in Fig. 5C, JNK phosphorylation was greatly increased in the C-MF group compared to that in the S-FGF2, S-MF, and negative control groups, while the level of total JNK remained unchanged. The activity of p65 showed a similar pattern, that is, a sharp increase in the level of phosphorylated p65 in the C-MF group compared to the other groups (Fig. 5D). Taken together, these data suggested that the FGF2 ligand in the form of a plate-coated MBP-FGF2 matrix could more strongly induce the signaling cascade related to IL-8 expression than other soluble ligands.

### IL-8 produced from FECS-Ad exerts angiogenic potential in vitro

**Fig. 6:**
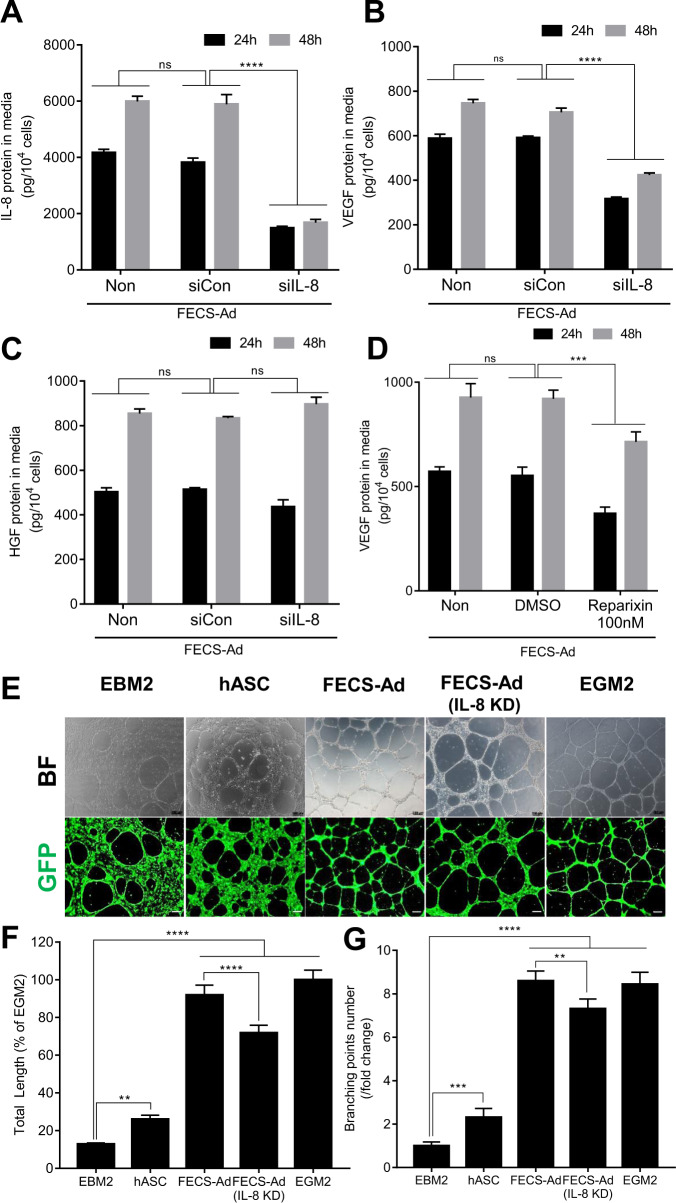
Effect of IL-8 from FECS-Ad on the angiogenic potential in vitro. **A**–**D** hASCs were treated with FGFR1 siRNA or reparixin, an IL-8 receptor inhibitor, and cultured on MBP-FGF2 surface to form FECS-Ad. Conditioned medium from FECS-Ad were prepared and analyzed by ELISA. **A** Knockdown efficiency of IL-8 protein in FECS-Ad. ns not significant, ****p < 0.0001 (two-way ANOVA), *n* = 3 per group. Effect of IL-8 knockdown on **B** VEGF and **C** HGF protein. ns not significant, ****p < 0.0001 (two-way ANOVA), *n* = 3 per group. **D** Effect of reparixin on VEGF protein. ns not significant, ***p < 0.001 (two-way ANOVA), *n* = 3 per group. **E**–**G** GFP-HUVECs plated on matrigel were co-cultured with hASCs, FECS-Ad, or IL-8 silenced FECS-Ad in transwell plate for 16 h. **E** Effect of hASC co-culture on the tube formation of HUVECs. **F** Effect on the total tube length of HUVECs. **p < 0.01, ***p < 0.001, ****p < 0.0001 (one-way ANOVA), *n* = 5 per group. **G** Effect on the number of branching points of HUVECs. **p < 0.01, ***p < 0.001, ****p < 0.0001 (one-way ANOVA), *n* = 5 per group. All data are presented as mean ± SD.

We further assessed the effect of IL-8 on the angiogenic potential of FECS-Ad in vitro by transfecting hASCs with IL-8 or control siRNA, followed by cell culture on the MBP-FGF2 surface for 24 or 48 h. FECS-Ad-conditioned media was prepared and followed by ELISA for expression of IL-8, VEGF, and HGF, which are highly induced during FECS-Ad formation. Transfection with IL-8 siRNA significantly reduced IL-8 secretion in FECS-Ad at both 24 and 48 h after attachment to the MBP-FGF2 matrix (Fig. 6A). VEGF expression was also decreased by IL-8 knockdown in FECS-Ad, suggesting that IL-8 might regulate the expression of VEGF in hASCs (Fig. 6B). However, the production of HGF was not affected by IL-8 silencing (Fig. 6C). To confirm autocrine regulation of IL-8 and VEGF in hASCs, cells were treated with 100 nM of reparixin, an IL-8 receptor inhibitor, and attached to the MBP-FGF2 substrate to generate FECS-Ad. Consistent with previous data, VEGF production was significantly decreased in FECS-Ad upon reparixin treatment (Fig. 6D).

To further investigate the effect of IL-8 from FECS-Ad on angiogenesis, tube formation of primary cultured human umbilical vein endothelial cells (HUVECs) was analyzed. HUVECs cultured on Matrigel plates are known to gradually form capillary-like tubular structures, which resemble blood vessels. As shown in Fig. 6E and Supplementary Fig. 5A, co-culture with monolayer hASCs rarely induced tube formation in HUVECs. However, co-culture with FECS-Ad significantly promoted the formation of tubular structures compared to the control (EBM2) (Supplementary Fig. 5B,C). Co-culturing with an increasing concentration of FEC-Ad regulated the degree of tube formation in a dose-dependent manner, measured by total tube length and number of branching points (Supplementary Fig. 5D–F). These data indicated that hASCs, as a form of 3D spheroid, might be able to induce angiogenesis of endothelial cells, presumably through secretory factors.

Next, the effect of IL-8 from FECS-Ad on HUVEC tube formation was tested by co-culturing GFP-HUVECs with monolayer hASCs, FECS-Ad, or IL-8-silenced FECS-Ad. FECS-Ad transplantation strongly induced the formation of HUVEC capillary structure, in comparison to control or monolayer hASCs (Fig. 6E). IL-8 silencing in FECS-Ad significantly decreased the total tube length and number of branching points of HUVECs compared to the FECS-Ad group (Fig. 6F, G). Overall, these data suggested that IL-8 produced from FECS-Ad might be involved in the process of in vitro angiogenesis of HUVECs.

### Effect of IL-8 on therapeutic potential of FECS-Ad in mouse hindlimb ischemia model

**Fig. 7:**
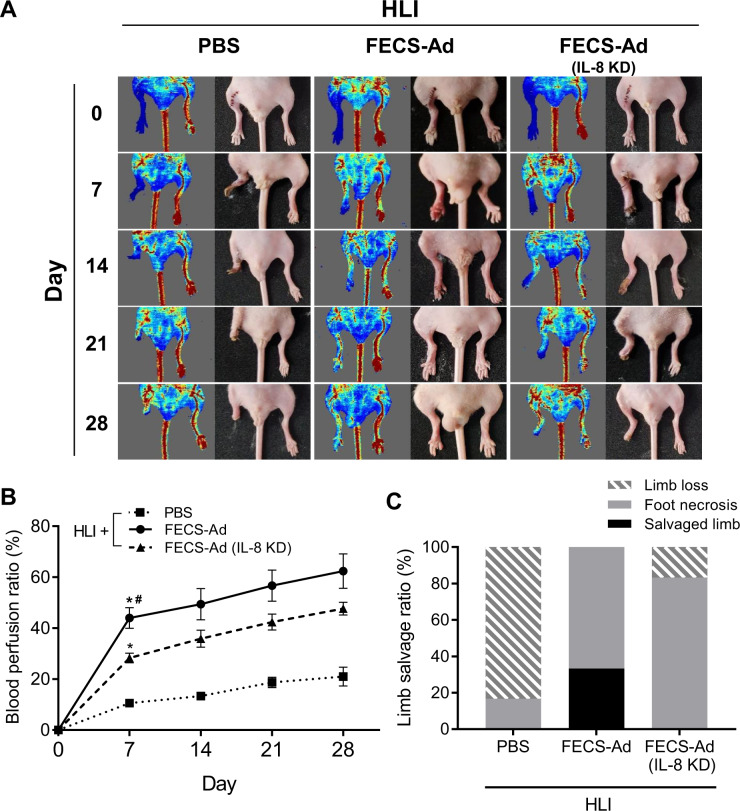
Roles of IL-8 in the therapeutic potential of FECS-Ad in mouse hindlimb ischemia. One day after HLI induction, mice were i.m. injected with PBS, FECS-Ad, or IL-8-silenced FECS-Ad. Morphometric analysis and blood perfusion was assessed at 7, 14, 21, and 28 days post-surgery. **A** Representative images of hindlimb morphology and blood perfusion of each group. *n* = 6 per group. **B** Blood perfusion ratio (percentage of normal limb) of ischemic limbs quantitated by laser Doppler imaging. Asterisk denotes significant difference compared to PBS group (*p* < 0.0001) and hash denotes significant difference compared to FECS-Ad (IL-8 KD) group (*p* < 0.0001) (two-way ANOVA). *n* = 6 per group. **C** Physiological status of ischemic limbs assessed at 28 days post-surgery. *n* = 6 per group. All data are presented as mean ± SEM.

We previously reported that implantation of 3D hASC spheroids formed using the MBP-FGF2 platform could efficiently promote angiogenesis and tissue regeneration in a mouse hindlimb ischemia (HLI) model^16^. To mimic the clinical scenario in which cell therapies for CLI are performed after ischemic conditions are established, PBS, FECS-Ad, or IL-8-silenced FECS-Ad was injected into the thigh muscle adjacent to the ligated femoral artery 1 day after surgery. Laser Doppler perfusion imaging revealed a dramatic decrease in blood flow in the ischemic limb shortly after femoral artery ligation (Fig. 7A). Follow-up demonstrated progressive recovery of blood flow with significantly higher post-ischemic hemodynamic recovery in FECS-Ad-treated mice compared to the PBS group. IL-8-silenced FECS-Ad injection reduced the blood flow ratio compared to the FECS-Ad group (Fig. 7B). Quantification of limb salvage was also performed 28 days post-surgery, with 66.7% and 33.3% of PBS-treated mice experiencing complete or severe limb loss, respectively. However, limb salvage and foot loss was achieved in 33.3% and 66.7% of FECS-Ad-treated mice, respectively. In the IL-8-silenced FECS-Ad group, none of the mice had salvaged limbs, while 83.3% and 16.7% of mice experienced foot and limb loss, respectively. These results suggested that exogenously delivered FECS-Ad might contribute to the recovery process after HLI and that IL-8 from FECS-Ad played an important role in restoring blood perfusion and attenuating necrosis of ischemic tissue.

### Effect of IL-8 on angiogenic potential of FECS-Ad in mouse hindlimb ischemia model

**Fig. 8:**
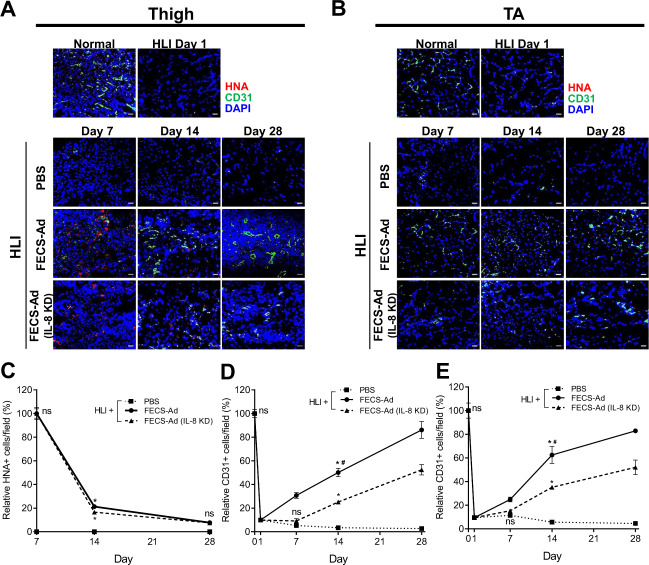
Roles of IL-8 in the angiogenesis of FECS-Ad in mouse hindlimb ischemia. Ischemic thigh and TA muscles isolated from PBS, FECS-Ad, or IL-8-silenced FECS-Ad-injected mice were immunostained with HNA and CD31. Nuclei were stained with DAPI. HNA and CD31 staining of **A** ischemic thigh and **B** TA of each group at 1, 7, 14, and 28 days post-surgery. *n* = 5 per group. Scale bar, 20 μm. **C** Ratio of HNA-positive cells in ischemic thigh. FECS-Ad group at day 7 was presented as 100%. ns not significant between FECS-Ad and FECS-Ad (IL-8 KD) group, *p < 0.0001 compared to PBS group (two-way ANOVA). *n* = 5 per group. Percentage of CD31-positive cells in **D** ischemic thigh and **E** TA. Normal group was presented as 100%. ns not significant between PBS and FECS-Ad group, *p < 0.0001 compared to PBS group, #*p* < 0.0001 compared to FECS-Ad (IL-8 KD) group (two-way ANOVA). *n* = 5 per group. All data are presented as mean ± SEM.

The effect of IL-8 on the angiogenic potential of FECS-Ad in HLI was also investigated. Femoral artery occlusion was induced, and PBS, FECS-Ad, or IL-8-silenced FECS-Ad was i.m. injected 1 day after surgery. Muscles in the treated thigh region and the tibialis anterior (TA) muscle were analyzed by immunofluorescence using antibodies against CD31 and HNA. Few CD31-positive cells were observed in the thigh muscles of ischemic limbs 1 day after surgery, prior to i.m. injection. In PBS-treated mice, CD31-positive cells were rarely observed until 28 days post-surgery (Fig. 8A). However, FECS-Ad treatment induced a dramatic increase in the population of CD31-positive cells, which was significantly reduced by IL-8 knockdown in FECS-Ad (Figs. 8A and 8D). A similar immunostaining pattern was observed in the TA muscle, suggesting that FECS-Ad injection might promote angiogenesis not only in the injected area, but also in the distal region of the ischemic limb (Fig. 8B, E). We further analyzed the density of microvessels and arterioles by immunofluorescence using an α-SMA antibody. Similar observations were made with the α-SMA-positive cell population, that is, a progressive increase in blood vessel density was observed in the FECS-Ad-injected group compared to the PBS-treated group. IL-8 silencing in FECS-Ad significantly lowered the α-SMA-positive cell ratio in both thigh and TA muscles (Supplementary Fig. 6A–D). Next, the distribution of hASCs from FECS-Ad after transplantation to the ischemic muscle was assessed by immunostaining for human nuclear antigen (HNA). FECS-Ad injected into the thigh muscle retained their spherical shape at day 3 (Supplementary Fig. 6E). HNA-positive cells were observed at day 7 in both the FECS-Ad and IL-8-silenced FECS-Ad groups (Fig. 8A), and their numbers gradually decreased until 28 days post-surgery. HNA-positive cell populations between the two groups did not differ significantly (Fig. 8C). HNA-positive cells were not observed in TA muscles in any of the groups, indicating that hASCs implanted in the form of FECS-Ad only existed in the local area of the injection site (Fig. 8B).

### Effect of IL-8 on tissue regeneration potential of FECS-Ad in mouse hindlimb ischemia model

**Fig. 9:**
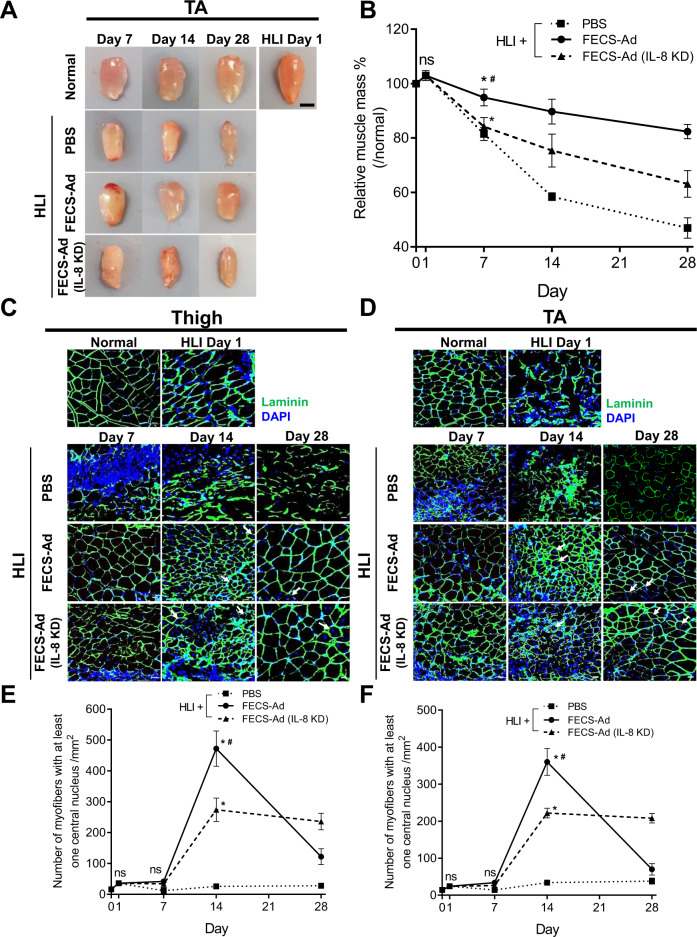
Roles of IL-8 in the tissue regenerative potential of FECS-Ad in mouse hindlimb ischemia. Ischemic thigh and TA muscles from PBS, FECS-Ad, or IL-8-silenced FECS-Ad-treated mice were prepared followed by immunofluorescence. **A** Representative TAs at appropriate times after ischemic surgery are shown in the photos. *n* = 4 per group. Scale bar, 2 mm. **B** Time kinetics of TA weights of each group 1, 7, 14, and 28 days after surgery. ns not significant among PBS, FECS-Ad, and FECS-Ad (IL-8 KD) group, *p < 0.0001 compared to PBS group, #*p* < 0.0001 compared to FECS-Ad (IL-8 KD) group (two-way ANOVA). *n* = 4 per group. Laminin staining of **C** ischemic thigh and **D** TA of each group at 1, 7, 14, and 28 days post-surgery. Nuclei were stained with DAPI. Scale bar, 20 μm. *n* = 5 per group. Quantification of muscle fibers with at least one centralized nuclei in **E** ischemic thigh and **F** TA. ns not significant among PBS, FECS-Ad, and FECS-Ad (IL-8 KD) group, *p < 0.0001 compared to PBS group, #*p* < 0.0001 compared to FECS-Ad (IL-8 KD) group (two-way ANOVA). *n* = 5 per group. All data are presented as mean ± SEM.

We further analyzed whether IL-8 produced from FECS-Ad affected tissue regeneration in the HLI model. As shown in Fig. 9A, B, TA muscle mass decreased in PBS-treated mice after HLI induction to 82%, 58%, and 47% of control muscle mass values at days 7, 14, and 28, respectively. When mice were injected with FECS-Ad, reduction of muscle mass was inhibited to 95%, 90%, and 82% of control muscle mass values at days 7, 14, and 28, respectively. However, IL-8 silencing in FECS-Ad disrupted this reversal of muscle weight reduction, resulting in a muscle mass decrease to 84%, 75%, and 63% of control muscle mass values at days 7, 14, and 28, respectively (Fig. 9B).

Muscle cross sections were analyzed by immunostaining with antibodies against laminin. Thigh muscles were isolated and the number of regenerating myofibers was quantified, characterized by centralized nuclei. Infiltration of mononucleated cells and slight myofiber disintegration were observed 1 day after surgery, implying the degeneration of muscle fibers by ischemia (Fig. 9C). In the PBS-treated group, few intact myofibers and fibers with centralized nuclei were observed until 28 days post-surgery, indicating that incomplete regeneration occurred (Fig. 9C). However, in FECS-Ad-injected mice, most damaged myofibers were cleared and replaced by an abundance of newly formed regenerating fibers 14 days after surgery. At 28 days after HLI induction, the number of myofibers with centralized nuclei was dramatically reduced, suggesting that muscle architecture was largely restored in the FECS-Ad group. In contrast, significantly fewer regenerating fibers were observed in mice treated with IL-8-silenced FECS-Ad 14 days after surgery compared to the FECS-Ad treated mice. In the IL-8-silenced FECS-Ad group, myofibers with centralized nuclei were observed 28 days post-surgery, indicating that muscle regeneration promoted by injection of FECS-Ad might be delayed by IL-8 knockdown (Fig. 9C, E). Similar observations were made in the TA muscle, suggesting that FECS-Ad treatment might promote tissue regeneration in the distal region of the ischemic limb, presumably by affecting angiogenesis (Fig. 9D, F). We also investigated overall tissue damage and repair of the ischemic limb by H&E staining. In accordance with the immunostaining pattern, H&E staining of thigh and TA muscles revealed extensive myofiber degeneration and mononuclear cell accumulation in PBS-injected mice. FECS-Ad treatment facilitated muscle regeneration after HLI induction, and IL-8 silencing impaired the regenerative potential of FECS-Ad, as measured by the increased number of centralized nuclei in muscle fibers compared with the FECS-Ad-treated mice (Supplementary Fig. 6F–G).

**Fig. 10:**
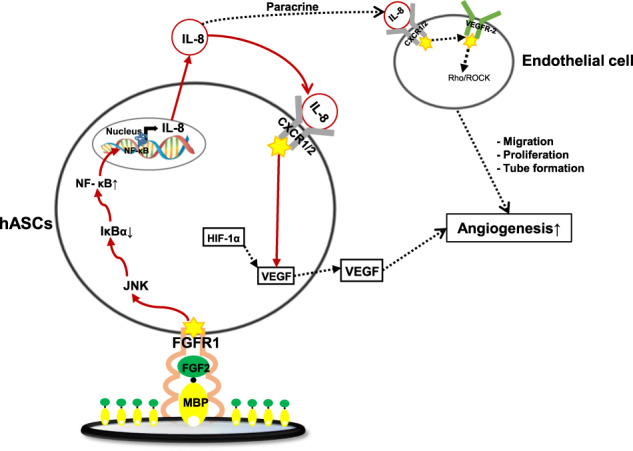
Schematic model of FECS-Ad mechanism of action for therapeutic angiogenesis. Proposed model for IL-8 production in FECS-Ad and its therapeutic potential in ischemic diseases. MBP-FGF2 binds to the FGFR1 of hASCs, activates JNK-NF-κB cascade to produce IL-8. IL-8 secreted from FECS-Ad not only acts on hASCs in an autocrine manner to induce VEGF secretion, but also contributes to the formation of tubular structure of endothelial cells. The red solid line is defined in this study, and the dotted line is already known through reference literature^16,37^.

Taken together, these data strongly indicated that mesenchymal stem cell delivery in form of 3D spheroids could promote angiogenesis and tissue regeneration to ameliorate ischemic conditions (Fig. 10).

## Discussion

In this report, we demonstrated that FECS-Ad generated from the novel MBP-FGF2 platform could exert a potent angiogenic effect on the mouse HLI model, and that IL-8 is a major regulator of FECS-Ad in therapeutic angiogenesis. IL-8 expression was greatly increased during the formation of FECS-Ad, and was regulated by the immobilized FGF2-mediated priming effect, rather than by hypoxia or 3D stimuli. MBP-FGF2 regulated the expression of IL-8 through the FGFR1/JNK/NF-κB axis. When cells were attached to the FGF2-immobilized surface, the activity of downstream signaling and IL-8 expression were increased more than when treated with soluble ligands. Exogenous delivery of FECS-Ad to the affected region by i.m. injection improved ischemic conditions by all measurements, including blood flow, limb salvage ratio, and tissue regeneration. Taken together, our data strongly suggest that FECS-Ad generated by a unique FGF2-immobilized substrate might effectively ameliorate critical limb ischemia in a mouse model by promoting various angiogenic factors. Various materials with immobilized bioactive molecules have been designed to improve cell functions. For example, leukemia inhibitory factor (LIF) was immobilized on a maleic anhydride copolymer to support pluripotency in mouse embryonic stem cells through intracellular signaling^28^. hASC spheroids generated using PDGF-immobilized mineral fibers presented an osteogenic phenotype and promoted bone regeneration when implanted^29^. Chemically and physically engineered surface materials to generate cell spheroids have several reported advantages as therapeutics. For example, poly (2-hydroxyethyl methacrylate) is a widely used cost-effective chemical reagent that prevents cell adhesion and induces 3D formation^30^. Biomaterials such as agarose are also used to generate multicellular tumor spheroids^31^. Here, we demonstrated that a MBP-FGF2-coated surface contributed to forming cell spheroids through control of cell-matrix adhesion and to priming hASCs through the FGF2-FGFR1 signaling cascade. Given the fact that the major limitations in stem cell therapy are low efficacy and cell viability, MBP-FGF2, as a single biomolecule, might have the potential to overcome both hurdles by affecting cell adhesion and priming mechanisms. It is interesting to note that the immobilized FGF2 substrate not only assisted cell-matrix interaction and formation of 3D spheroids, but also transduced the FGF2 signaling cascade more efficiently than soluble FGF2 ligands. HSPG is known to act as a low-affinity binding site for FGF2, and also function as a modulator of FGF2 activity. HSPG can bind to the FGF2- FGFR1 complex, stabilize ligand binding to its receptor, and potentiate receptor dimerization and signaling^23^. In accordance with our work, recent studies have demonstrated that growth factors present in the solid phase could more effectively trigger cellular responses compared to soluble administration^32,33^. In the solid phase, growth factors tethered to surfaces are spatially confined and have limited diffusion. Further, adhesion receptors can be clustered along the growth factor-coated surface, thereby enhancing signaling^34^. It remains to be elucidated whether MBP-FGF2 would also induce clustering of FGFR1, but our results suggest that the MBP-based growth factor tethering system could be applied to boost signals in various cell types according to several situations. In this study, IL-8 suppression in FECS-Ad inhibited angiogenic effect not only in vitro, as determined by tube formation of HUVEC cells, but also in vivo, as measured by blood flow in the murine HLI model. To efficiently reduce IL-8 expression in FECS-Ad, we adapted a widely used liposome-based siRNA delivery methodology. The use of a transient knockdown system rather than a persistent knock-out technology to assess long-term therapeutic effects may be controversial. However, IL-8 expression induced during FECS-Ad formation gradually decreased after 3D formation, reaching basal level after 3 days and it is well understood that silencing gene expression by siRNA can be rescued within 2–3 days after transfection^35^. Expression of IL-8 in FECS-Ad is likely mediated by FGF2 signaling, which was reduced when cells were detached from the MBP-FGF2 matrix. Our data also demonstrated that single knockdown of IL-8 in FECS-Ad inhibited the therapeutic effect of FECS-Ad even 4 weeks after inducing ischemic conditions. Further, a small portion of the implanted cells, which were HNA-positive, expressed CD31 7 and 14 days after surgery, indicating that transplanted FECS-Ad might differentiate into the endothelial lineage and become incorporated into the host vasculature. Our research group previously reported that 3D-cultured hASCs could express the endothelial phenotype in vitro^36^, and IL-8 is well-documented for its roles in proliferation, migration, and survival of endothelial cells^37^. Therefore, IL-8 in hASCs might contribute to the endothelial phenotyping of transplanted cells, and FECS-Ad might exert angiogenic effects both by incorporating directly into neovessels and by secreting paracrine factors. Further studies are warranted to clarify these results, since it remains unknown whether transplanted human adult stem cells could be directly incorporated into new blood vessels. Overall, these results indicate that IL-8 produced from FECS-Ad would play an indispensable role(s) in the diverse recovery stages of ischemia. IL-8 was first discovered as a potent chemotactic agent for neutrophils and macrophages, and was later found to also contain angiogenic activity. This chemokine is released early post-injury mainly by macrophages, and plays a key role in the recruitment of neutrophils and other immune cells to the damaged site^38^. Therefore, IL-8 produced from FECS-Ad might potentially participate in the infiltration of immune cells, thereby regulating tissue regeneration. Further studies are in progress to clarify whether FECS-Ad could exert an immunomodulatory effect. Several reports have demonstrated that IL-8 is strongly involved in the process of angiogenesis; however, these studies have mainly investigated tumor development^39^. IL-8 secreted from tumor-infiltrating macrophages induces proliferation and migration of endothelial cells in the tumor microenvironment^39^. To our knowledge, the role(s) and therapeutic potential of IL-8 in ischemic tissues had not yet been elucidated, despite its interesting biological characteristics. Current treatment methods for ischemic diseases are extremely limited, and their efficacy, if any, is marginal^40^. No cell-based products have been approved for use in CLI treatment^40^, supporting the relevance of our study findings. In summary, FECS-Ad appears to exert potent therapeutic effects in ischemic tissue recovery by secreting angiogenic factors, including IL-8. Treatment with FECS-Ad not only improved blood flow in ischemic tissues, but also promoted muscle tissue regeneration. Therefore, FECS-Ad may produce multiple positive effects in ischemic diseases associated with tissue necrosis. Further studies are warranted to investigate the possibility of using this FECS-Ad, and in particular, functionalized 3D stem cells, for treatment of various ischemic diseases. Our study findings provide novel insight into the role(s) of IL-8 in blood vessel recovery and tissue repair during ischemia. FECS-Ad and MBP-FGF2 may contribute to the development of stem cell therapeutics based on bio-functional materials for the treatment of CLI.

## Methods

### Preparation of MBP-FGF2 matrix on microwell plates

Recombinant MBP-FGF2 protein was produced from the *E. coli* vector as previously described^41^. Briefly, human FGF2 cDNA sequence (Bioneer, Seoul, Korea) were cloned to pMAL-c2X vector (vector containing MBP coding sequence) (New England Biolabs, MA), and transfected to the *E. coli* strain BL21 (DE3) (New England Biolabs, MA). *E. coli* harboring pMAL-FGF2 was cultivated in Luria-Bertani (LB) broth (ThermoFisher Scientific, MA) under agitation (200 rpm) to an OD_600_ (optical density at 600 nm wavelength) of 0.6. Protein expression was induced for 16 h at 25 °C with 1 mM Isopropyl β-d-1-thiogalactopyranoside (IPTG). The expressed MBP-fused FGF2 protein was eluted and purified according to manufacturer’s instruction (New England Biolabs, MA). 50 μL of MBP-FGF2 protein was dissolved in phosphate-buffered saline (PBS) to a final concentration of 20 μg/mL, and added to each well of 384-well microplates (Corning, NY). Excessive protein solutions were then removed from well by washing three times with PBS after 4 h at room temperature.

### hASC culture and FECS-Ad formation

hASCs (CEFO, Seoul, Korea) were grown in CEFOgro media (CEFO, Seoul, Korea) according to manufacturer’s instruction. Fifth passage (P5) hASCs were used for all experiments. For 3D spheroid (FECS-Ad) formation of hASCs, cells were trypsinized and suspended in StemPro® MSC serum-free media (SFM) (Gibco, MA), and plated with a density of 1 × 10^4^ cells per well in MBP-FGF2 coated 384-well microplates. Cells were cultured for 24 h at 37 °C incubator with 5% CO_2_. Formation of FECS-Ad was determined using an Axio Vert A1 phase contrast microscope (Zeiss, Oberkochen, Germany).

### HUVEC culture and tube formation assay with transwell

GFP-labeled human umbilical vein endothelial cells (Angio-Proteomie, MA) were cultured in EGM-2 media (Lonza, Basel, Switzerland) with provided supplementations and antibiotics (100 U/mL penicillin and 100 μg/mL streptomycin (Sigma Aldrich, MO)). For in vitro tube formation assay, lower compartment of pre-chilled 24-well transwell culture plates (Corning, NY) were coated with 250 μL per well of basement membrane extract (GFR; Growth Factor Reduced, Corning, NY) and incubated for 40 min at 37 °C. GFP-HUVECs were serum starved in EBM-2 media (Lonza, Basel, Switzerland) for 24 h, and seeded in GFR-coated plate at 1 × 10^5^ cells per well. Cells were incubated in 37 °C for 1 h for the attachment to the basement membrane. Then, FECS-Ad, or monolayer hASCs were additionally incubated to the upper compartment of transwell plates. After 16 h of co-culture, the tube formation morphology of GFP-HUVECs was analyzed with phase contrast microscope and confocal microscope (Zeiss, Oberkochen, Germany). Total length and the number of branching points of HUVEC tubes were evaluated using Image J software (National Institutes of Health, MD).

### EV isolation and nanoparticle tracking analysis

For the isolation of EVs, 10 mL of conditioned media from each group was collected after 24 h, filtered through a 0.22 μm polyethersulfone membrane (Millipore, MA) to remove cells and debris, and ultracentrifuged at 28,000×*g* for 90 min at 4 °C under vacuum (CP100NX, Eppendorf Himac Technologies, Hamburg, Germany). The supernatant was discarded and the pellet was resuspended in PBS. nanoparticle tracking analysis (NTA) was performed on samples in quintuplicate using the Malvern NanoSight NS300 (Malvern Instruments, Malvern, UK). The instrument was equipped with a red (642 nm) laser and sCMOS camera to estimate size distribution and particle concentration.

### Animal cares

Six-week-old male BALB/c nude mice were purchased from Orient Bio Inc. (Seongnam, Korea) for animal studies. Mice were housed at 24 °C with a 12 h light–dark cycle. All experiments were performed in compliance with the guideline set by the International Animal Care and Use Committee at Korea Institute of Science and Technology [KIST-2020-035].

### Surgical procedures

All surgical protocols were approved by the International Animal Care and Use Committee at Korea Institute of Science and Technology. Six-week-old male BALB/c nude mice were anesthetized with xylazine (10 mg/kg) and ketamine (100 mg/kg), and subjected to unilateral hindlimb surgery. The femoral artery of the right leg was excised gently from the proximal branch of the external iliac artery to the distal point where it bifurcates into the saphenous and popliteal arteries. Severed femoral artery endings were ligated with 5-0 black silk suture (AILEE, Pusan, Korea), and the incision was sutured using 5-0 silk suture. Blockade and recovery of hindlimb blood flow was monitored by Laser Doppler imaging (LDI) (Moor instruments) for up to 28 days after surgery.

### Cell administration

One day after arterial dissection, the mice were divided randomly into 3 experimental groups (*n* = 6 in each group). The control group received an injection of PBS (PBS group). Fifty numbers of FECS-Ad or IL-8 silenced FECS-Ad (5 × 10^5^ cells suspended in 0.2 mL of PBS per mouse) formed in 384-well microplate coated with MBP-FGF2 matrix were harvested and loaded to 1 mL syringe with 23-gauge needle (Kovax, Seoul, Korea). Cell administration was performed as previously described^15,16^. Briefly, the equal amount of cells was injected intramuscularly (i.m.) into the three locations (gracilis, adductor, and pectineal muscles) in the medial thigh of the ischemic limb.

### Immunohistochemistry and Morphometric analysis

For immunofluorescence staining, TAs and thigh muscles were harvested and fixed in 4% paraformaldehyde in PBS and frozen in optimal cutting temperature compound (Leica, Wetzlar, Germany) to be cut into 6 μm thickness. Sections were washed in 0.1 M PBS twice, and blocked with 5% fetal bovine serum (Gibco, MA), 2% bovine serum albumin (BSA) solution (Bovogen, Melbourne, Australia) mixed with 0.1% Triton X-100 in PBS for 1 h. Samples were incubated with primary antibodies diluted in blocking buffer overnight at 4 °C. The sections were then washed with PBS for 4 times and incubated with corresponding fluorescence-conjugated secondary antibodies (Invitrogen, CA), diluted in PBS for 1 h at room temperature. DAPI (Sigma Aldrich, MO) was used for nuclear staining. Fluorescence images were taken using Zeiss LSM 700 confocal microscope (Zeiss, Oberkochen, Germany), and analyzed through Image J software (National Institutes of Health, MD). Antibody information was included in Supplementary table 1B. For histological analysis, TAs and thigh muscles were fixed in 4% paraformaldehyde and dehydrated in gradient levels of ethanol followed by paraffin embedding as previously described^42,43^. Briefly, paraffin sections with 6 μm thickness were deparaffinized and rehydrated followed by staining with hematoxylin and eosin (H&E) (Sigma Aldrich, MO) according to standard protocols. Images were obtained using phase contrast microscope microscope (Zeiss, Oberkochen, Germany) to examine the morphology of the muscle.

### RNA isolation and RT-qPCR

Cells were prepared and mechanistically homogenized using Mini-Beadbeater-24 (Biospec Products, OK), and total RNA was extracted from cultured cells with RNeasy mini kit (Qiagen, Hilden, Germany) following the manufacturer’s instructions. One microgram of RNA was converted to cDNA using Superscript VILO cDNA synthesis kit (Invitrogen, CA) according to the manufacturer’s protocol. Gene expression was assessed using quantitative real-time PCR with ABI 7500 Real-Time System (Applied Biosystems, CA) and SYBR Premix Ex Taq (Takara, Kusatsu, Japan). Primer sequence information was included in the Supplementary Table 1A.

### ELISA

Cultured cell supernatants or cell lysates were prepared and subjected to hIL-8, hVEGF, and hHGF ELISA (R&D systems, MN) following the manufacturer’s protocol. To prepare cell lysates, cultured cells were harvested and mechanistically homogenized using Mini-Beadbeater-24 (Biospec Products, OK) and total proteins were extracted in RIPA lysis buffer (Sigma Aldrich, MO) containing a protease and phosphatase inhibitor cocktail (Abcam, Cambridge, UK). Samples were centrifuged at 12,000 rpm for 15 min at 4 °C and the supernatants containing total protein were used for ELISA.

### Western blot

For immunoblotting, cultured cells were prepared and homogenized in RIPA lysis buffer (Sigma Aldrich, MO) containing a protease and phosphatase inhibitor cocktail (Abcam, Cambridge, UK), using Mini-Beadbeater-24 (Biospec Products, OK). Equal amounts of protein were then separated by 4-15% gradient gel (Bio-Rad Laboratories, CA) and electrophoretically transferred to polyvinylidene fluoride membranes (Millipore, MA). The membranes were blocked with 5% BSA (Gibco, MA) in TBST (1 M Tris-HCl, pH 7.4, 0.9% NaCl and 0.05% Tween-20) for 1 h and probed with antibodies diluted in 3% BSA blocking solution overnight at 4 °C. Membranes were then incubated with HRP-conjugated anti-mouse or anti-rabbit IgG (1: 100,000; Sigma Aldrich, MO) for 1 h, and the protein bands were visualized with the enhanced chemiluminescence system (ThermoFisher Scientific, MA), and the images were taken using iBright CL1500 imaging system (ThermoFisher Scientific, MA). Antibody information was included in Supplementary table 1B.

### siRNA transfection

hASCs were transfected with siRNA specific to IL-8 (sc-39631, Santa Cruz Biotechnology, TX), EXT1 (sc-106792, Santa Cruz Biotechnology, TX), FGFR1 (4390825, Ambion™, MA) or scramble siRNA (sc-37007, Santa Cruz Biotechnology, TX) using RNAiMAX (ThermoFisher Scientific, MA) according to the manufacturer’s protocol. Briefly, hASCs were plated at 1.5 × 10^5^ cells per well in six-well culture plates. 24 h later, 25 pmol (2.5 μL, 10 μM) of IL-8, EXT1, FGFR1, or scramble siRNA was diluted into 125 μL of Opti-MEM (Gibco, MA), and 5 μL of RNAiMAX was diluted in 125 μL of Opti-MEM. Diluted siRNA and RNAiMAX were then combined and incubated at room temperature for 5 min. Subsequently, 250 μL of the siRNA-RNAiMAX mixtures were added to each well of a six-well plate. 24 h after transfection, cells were subjected to the analysis. Knockdown efficiency was evaluated by RT-qPCR or ELISA.

### Reagents

Recombinant human FGF2 protein (R&D systems, MN), PD173074 (Sigma Aldrich, MO), and anacardic acid (Abcam, Cambridge, UK) was used at appropriate concentrations. LY294002, U0126, SP600125, and SB203580 (Sigma Aldrich, MO) were used at 10 μM for experiments. Reparixin (Sigma Aldrich, MO) was used at 100 nM for experiments.

### Statistical analysis

All values are represented as mean ± SD or ± SEM from three or more independent experiments. Statistical significance was determined using two-way ANOVA, one-way ANOVA followed by Bonferroni’s multiple comparison tests or unpaired Student’s *t* test, provided by the GraphPad Prism 7 (GraphPad, CA) software.

### Reporting summary

Further information on research design is available in the Nature Research Reporting Summary linked to this article.

## Supplementary information

Supplementary Information

Supplementary Movie 1

Reporting Summary

## Data Availability

Datasets generated and/or analyzed during this study are available from the corresponding author on reasonable request.
