# Supplementary material for: FGF2-primed 3D spheroids producing IL-8 promote therapeutic angiogenesis in murine hindlimb ischemia
Source: NPJ Regen Med. 2021 Aug 18;6:48. doi: 10.1038/s41536-021-00159-7 (PMC8373896; doi:10.1038/s41536-021-00159-7)
Supplement: Supplementary file 1 — Supplementary Information [file 41536_2021_159_MOESM1_ESM.pdf]

Supplementary Figure 1

A

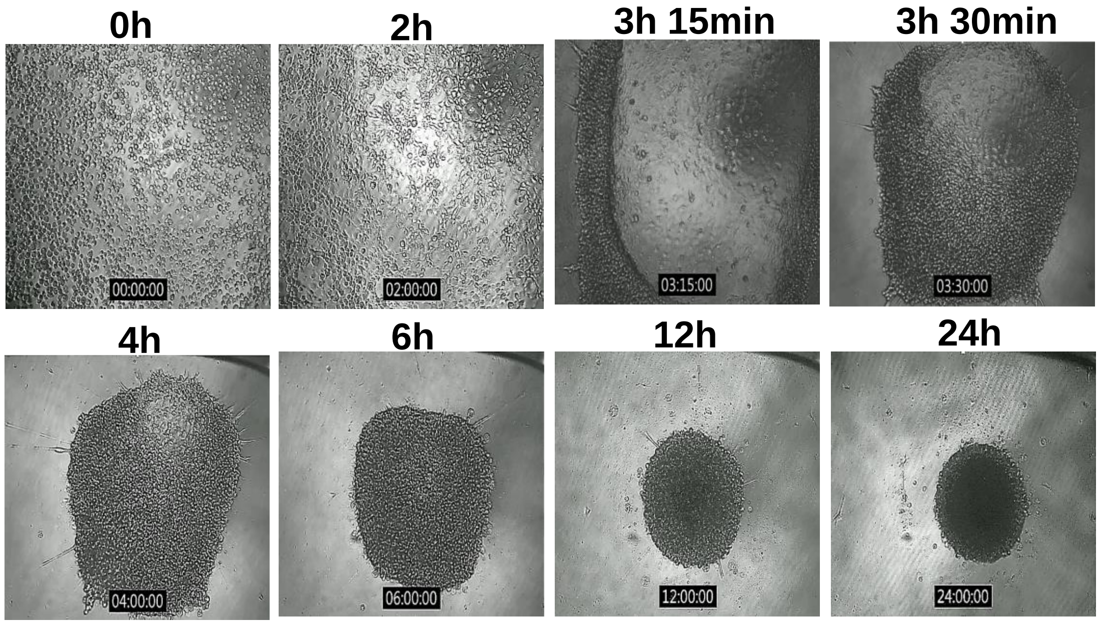

B

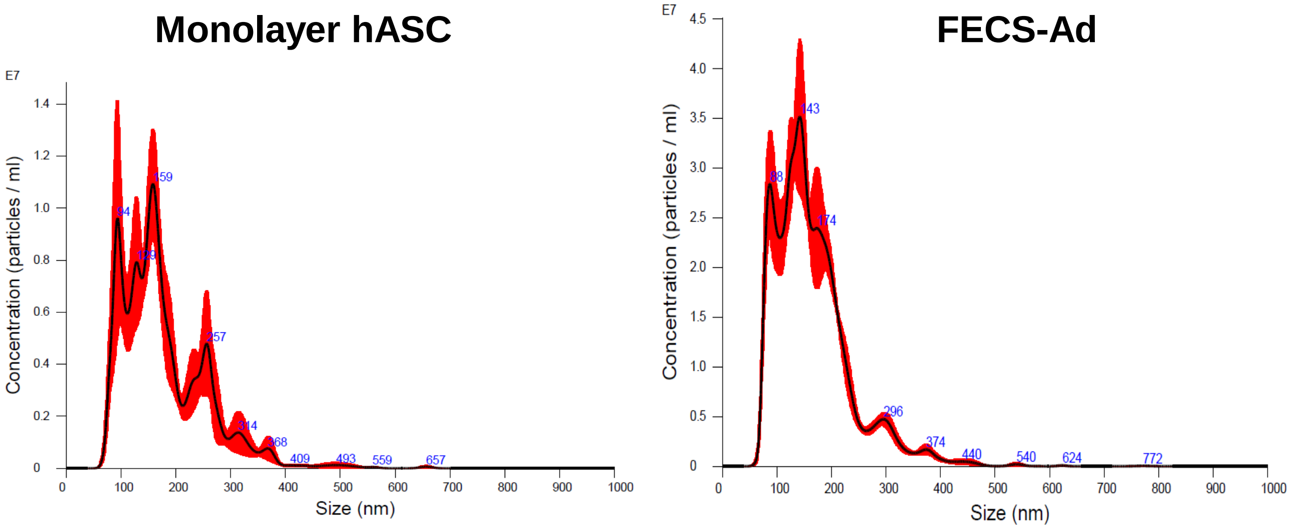

	Monolayer hASC	FECS-Ad
**Mean size**	179.1 ± 8.9 nm	165.2 +/- 1.5 nm
**Concentration**	$1.30 \times 10^9 \pm 9.70 \times 10^7$ particles/mL	$4.36 \times 10^9 \pm 1.80 \times 10^8$ particles/mL

C

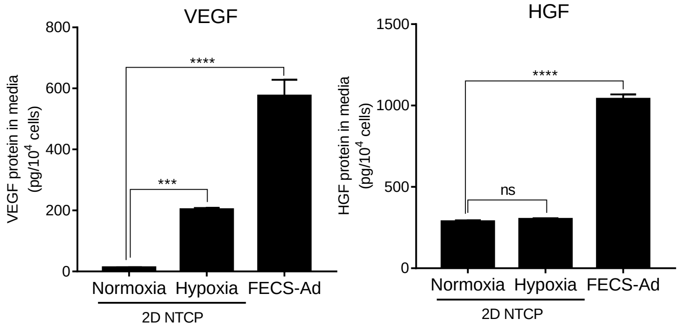

D

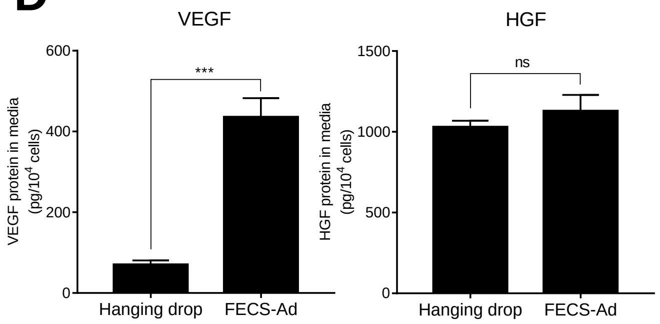

Supplementary Figure 2

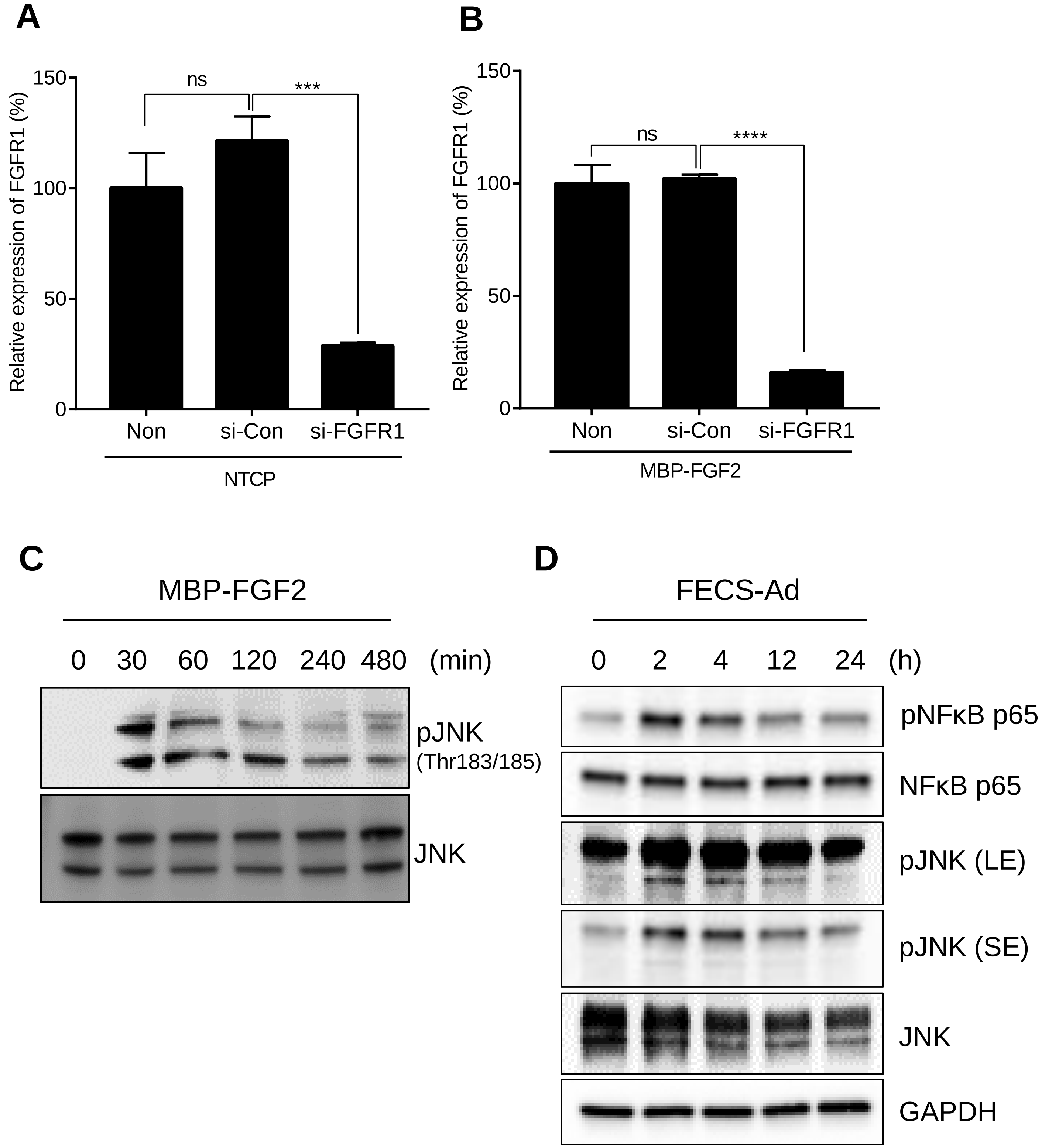

Supplementary Figure 3

A

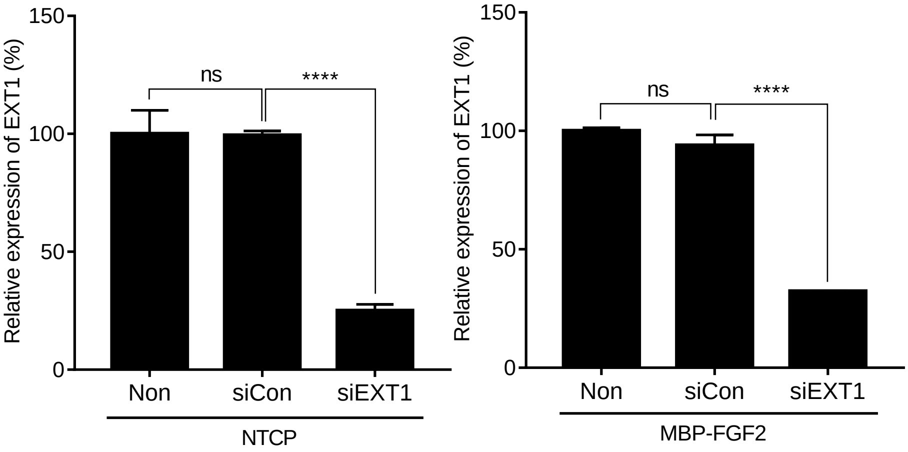

B

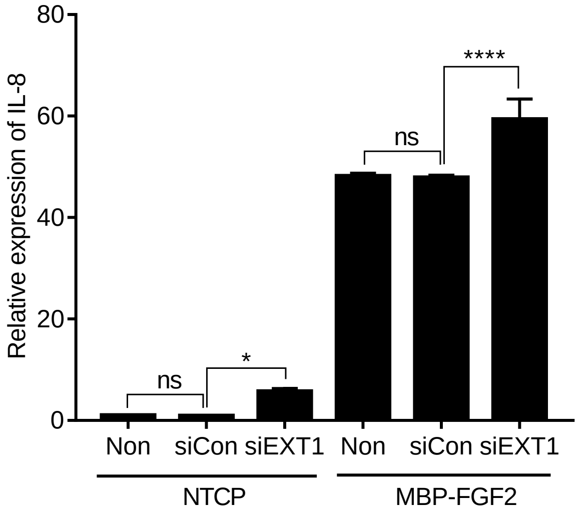

C

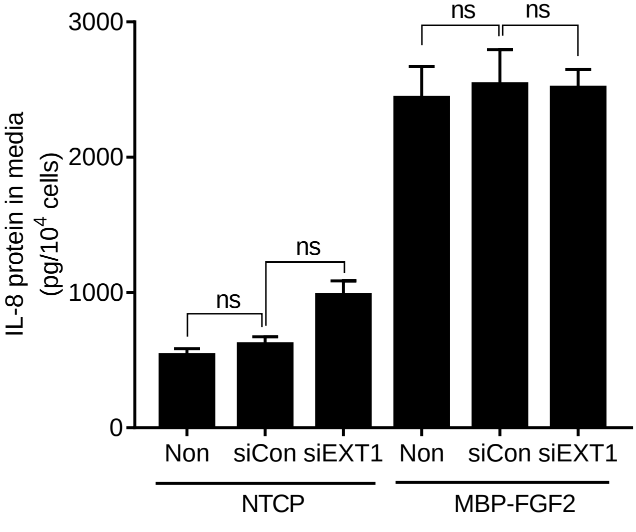

Supplementary Figure 4

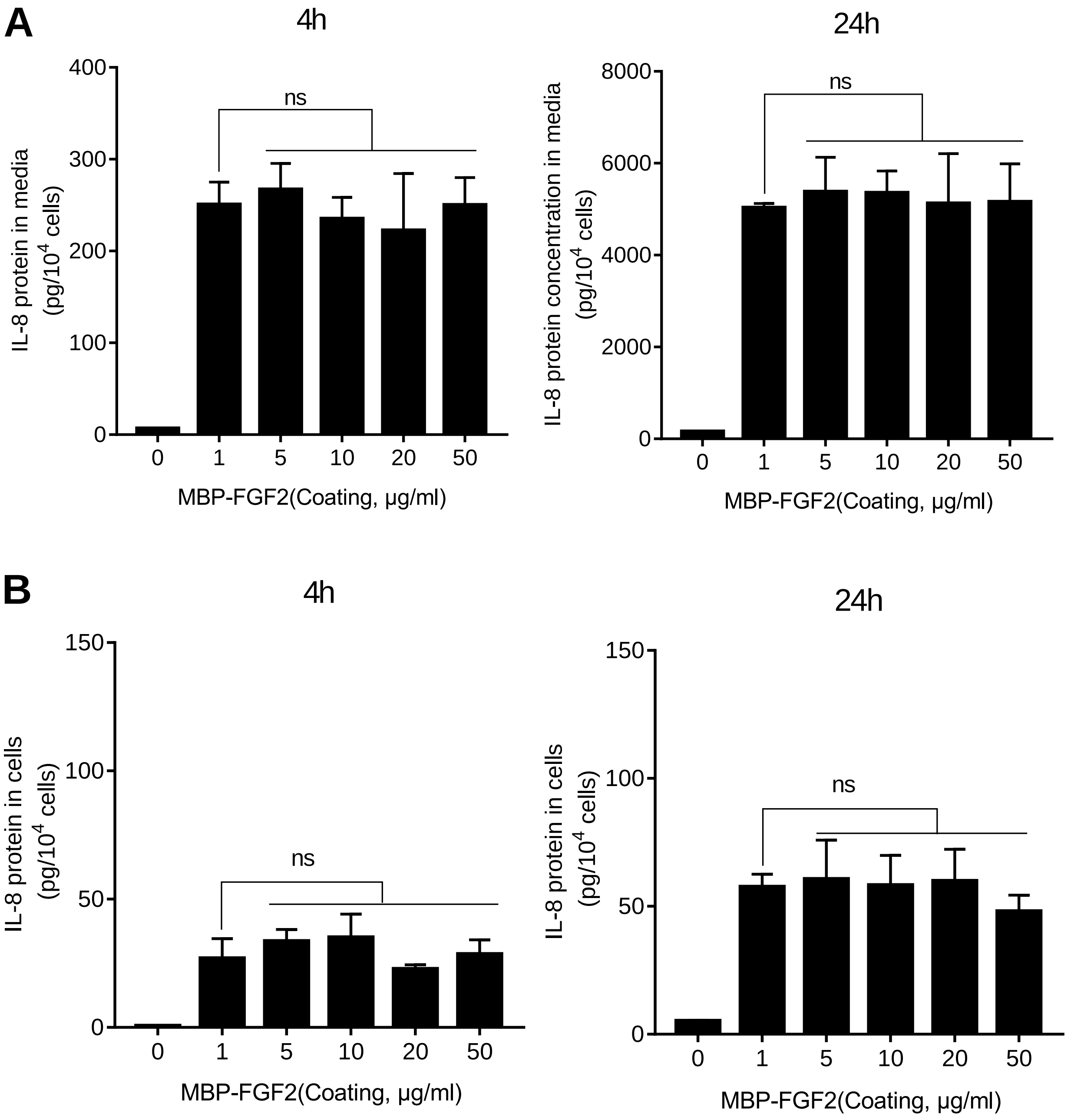

Supplementary Figure 4-Continued

C

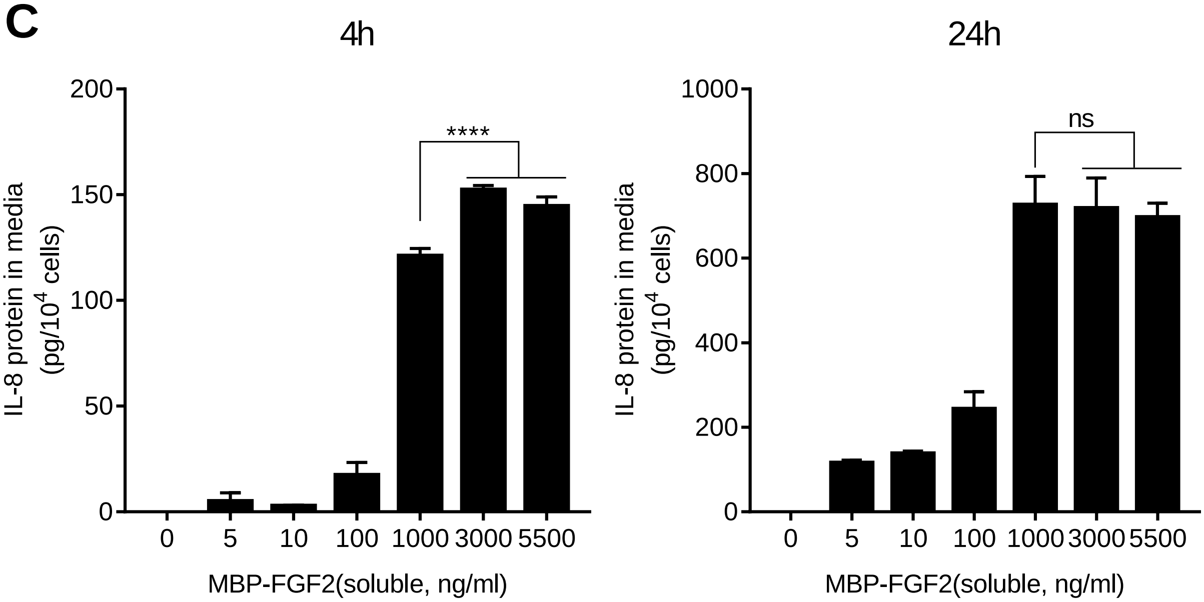

D

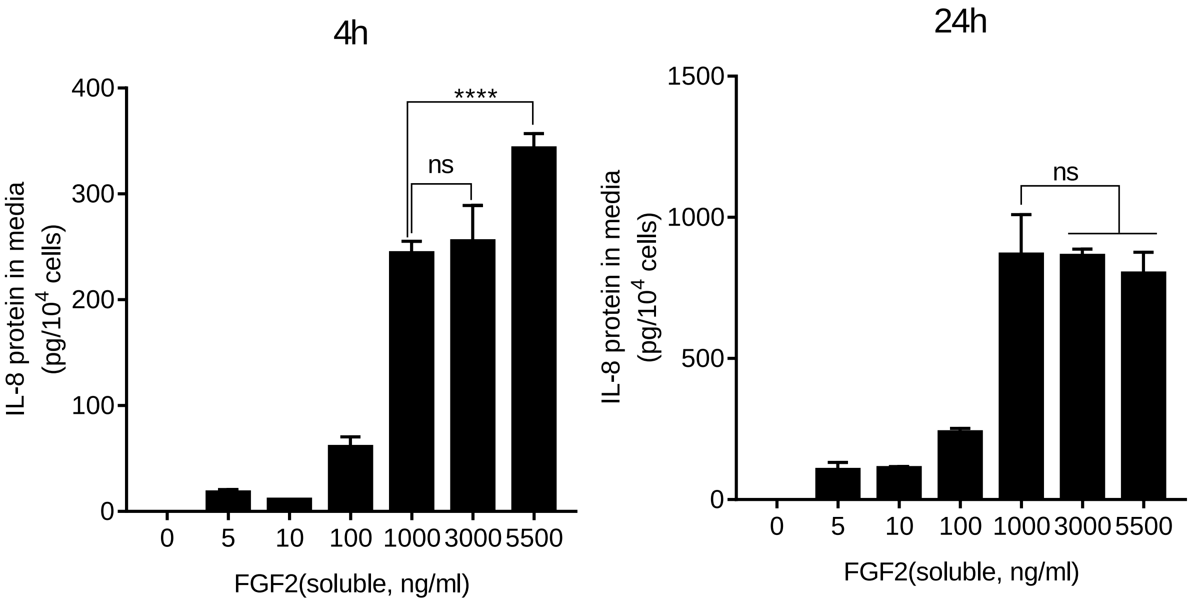

Supplementary Figure 5

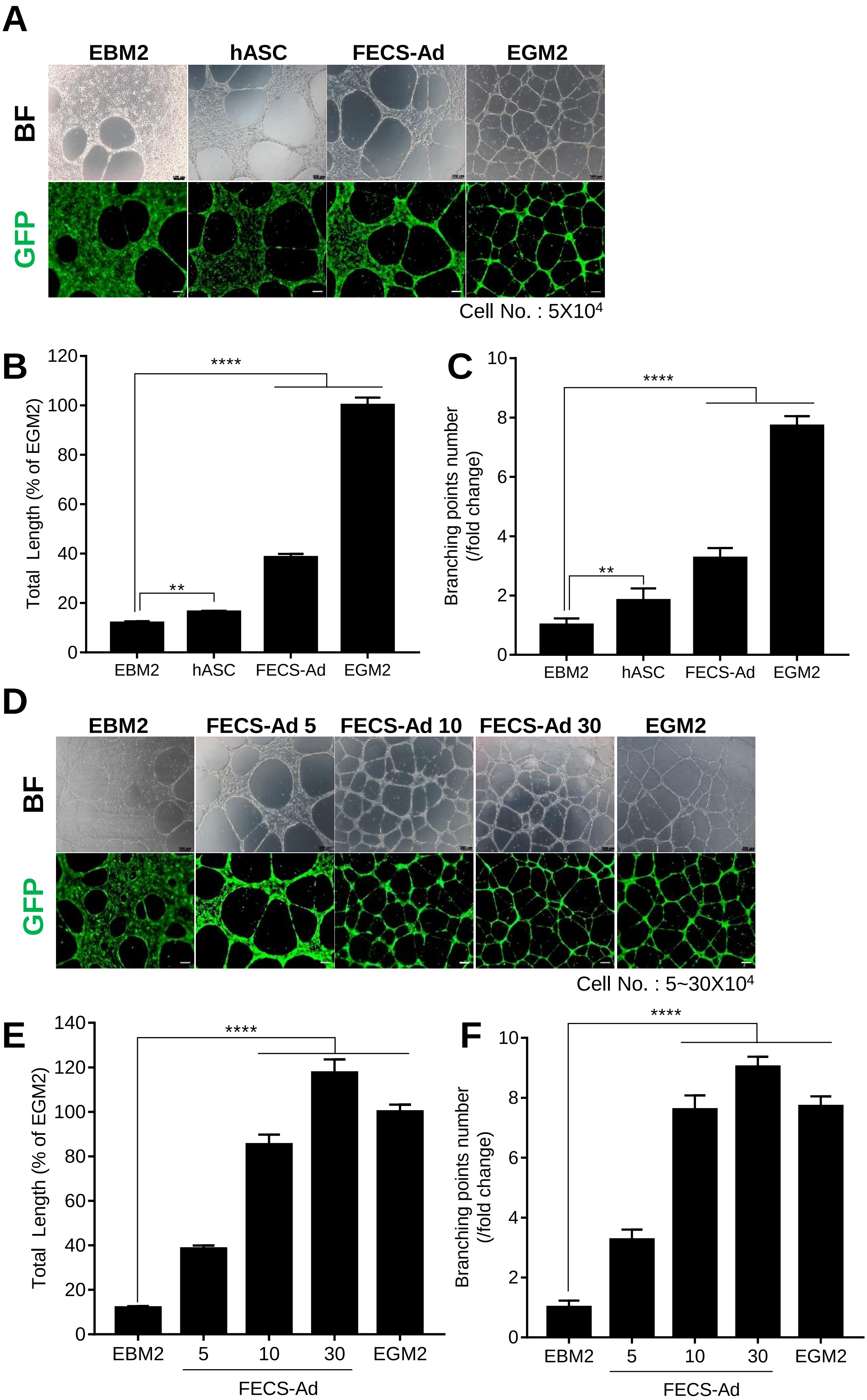

Supplementary Figure. 6

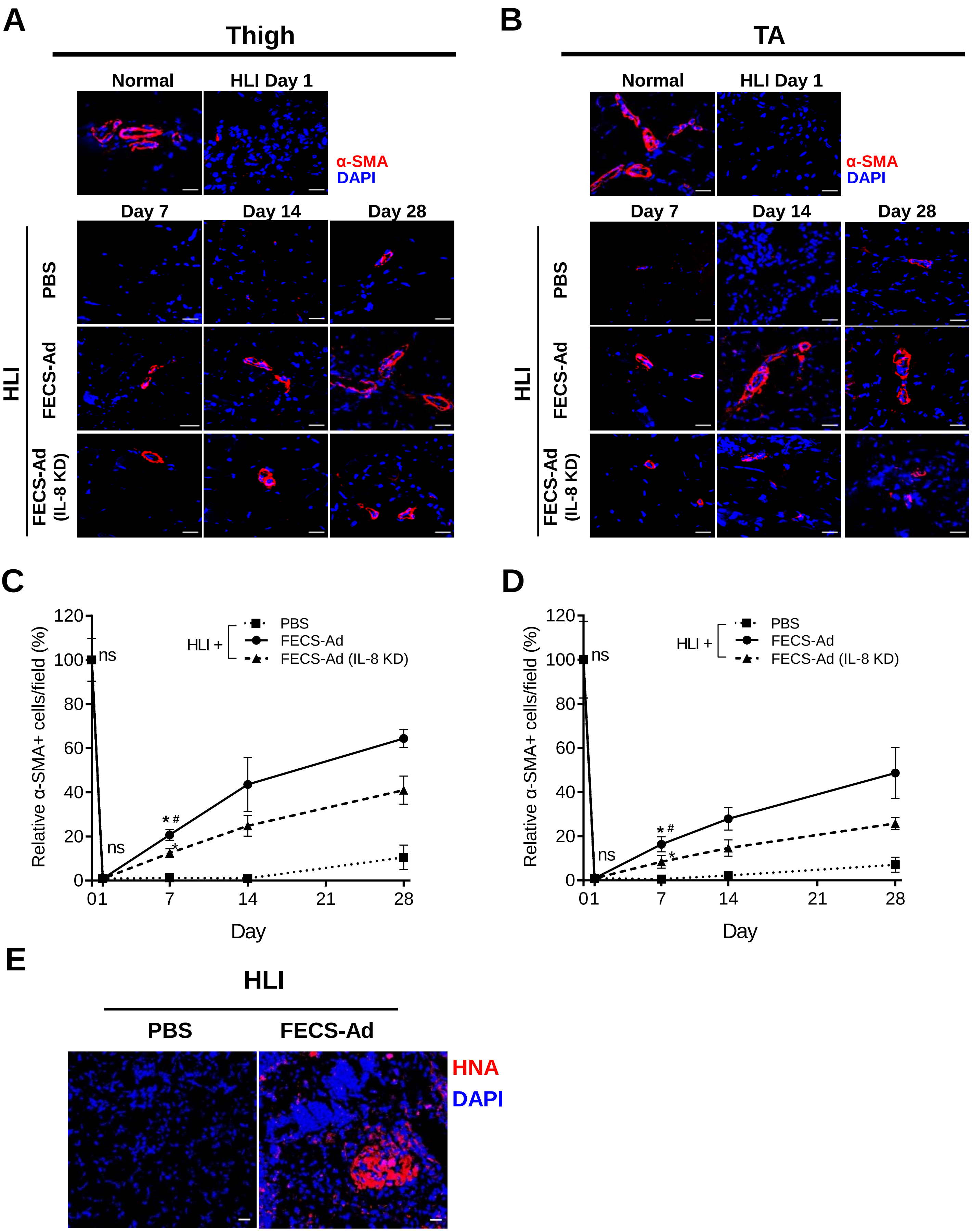

Supplementary Figure 6-Continued

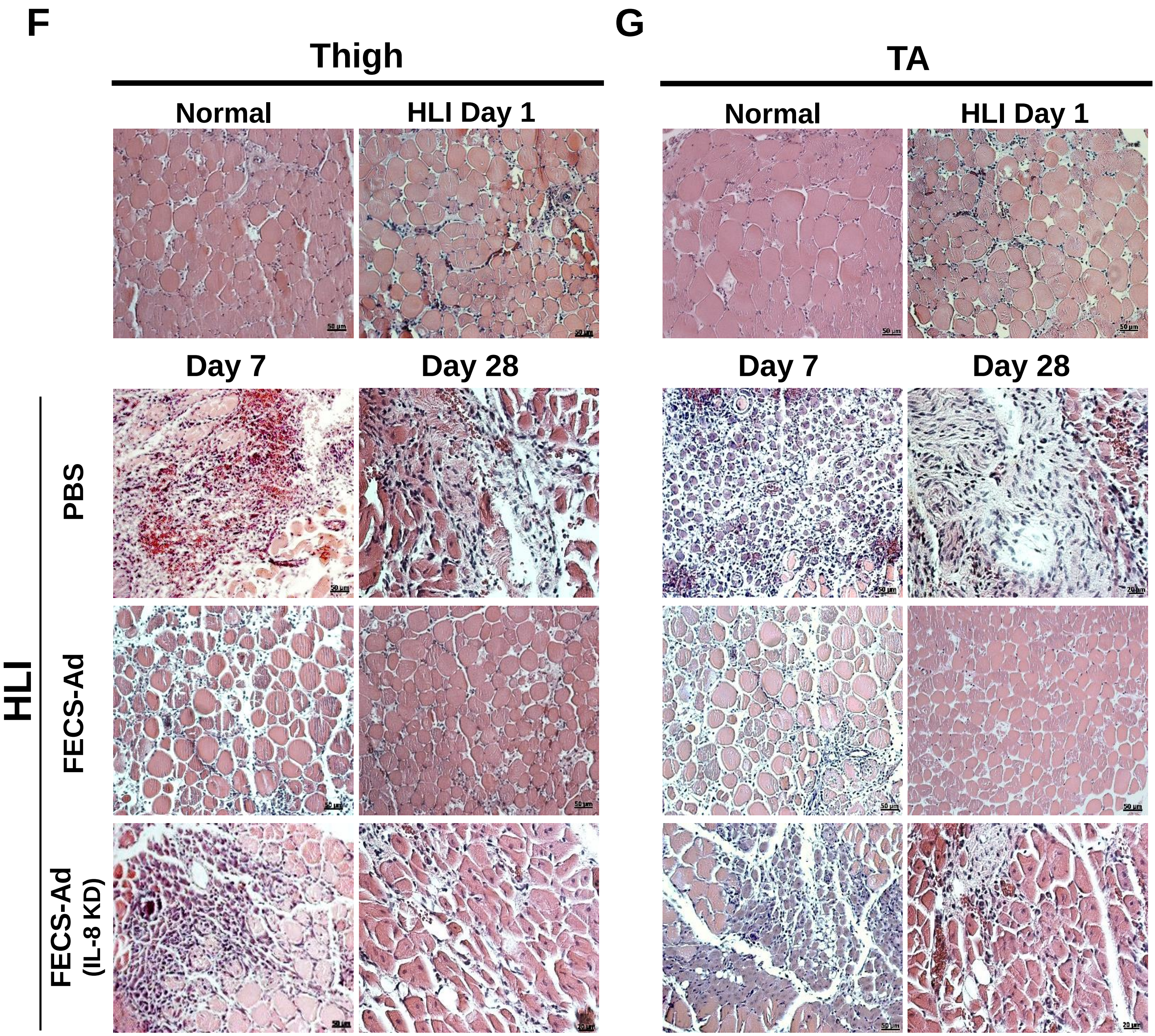

Supplementary Figure 7

Figure 4A_IκBα

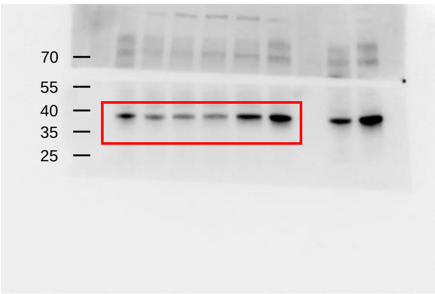

Figure 4A_pp65 (Ser536)

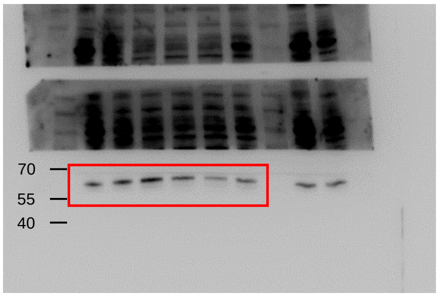

Figure 4A_p65

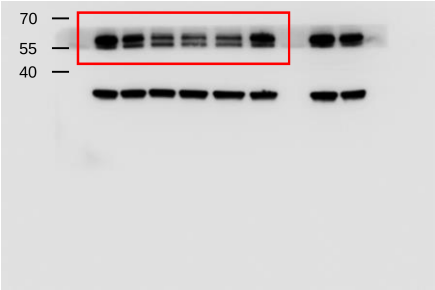

Figure 4A_β-actin

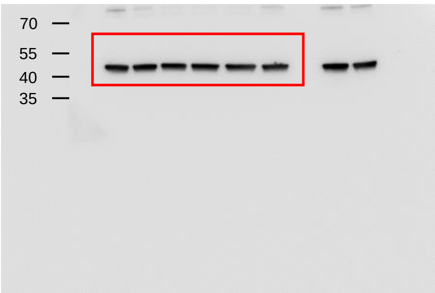

Figure 4C_pp65 (Ser536)

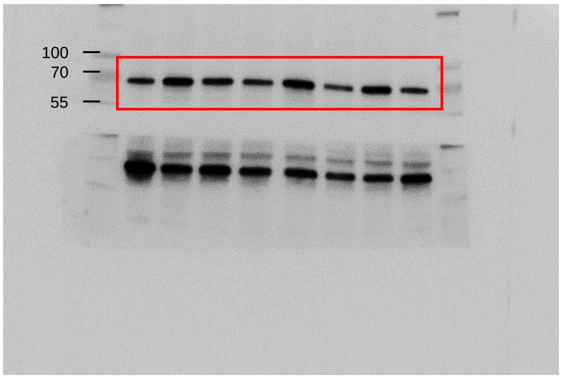

Figure 4C_p65

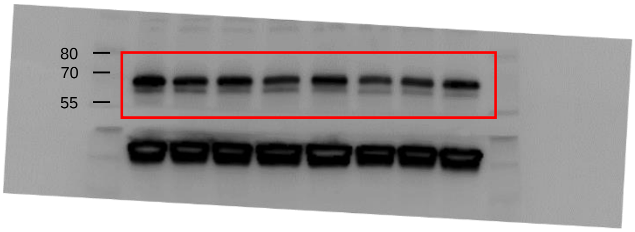

Figure 4C_β-actin

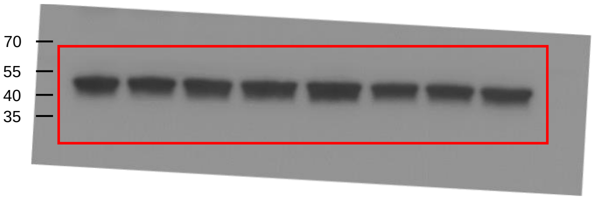

Supplementary Figure 8

Figure 5C_pJNK

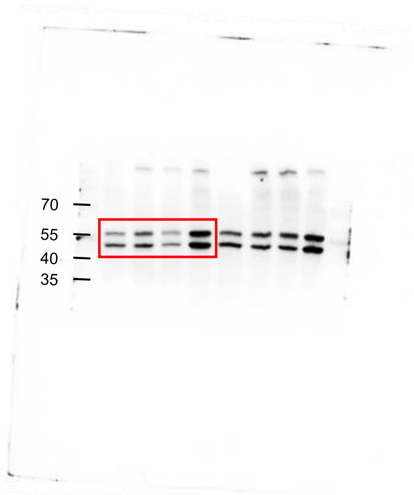

Figure 5C_JNK

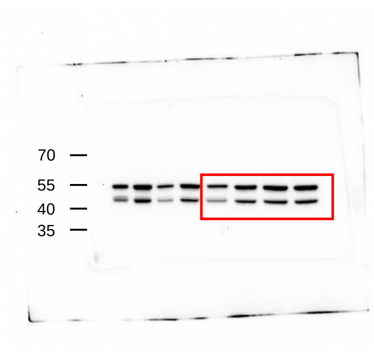

Figure 5C_β-actin

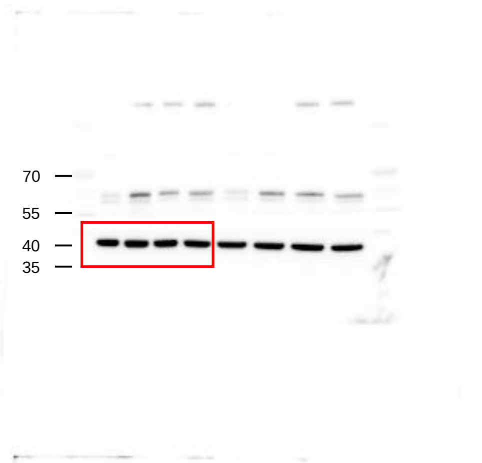

Figure 5D_pp65 (Ser536)

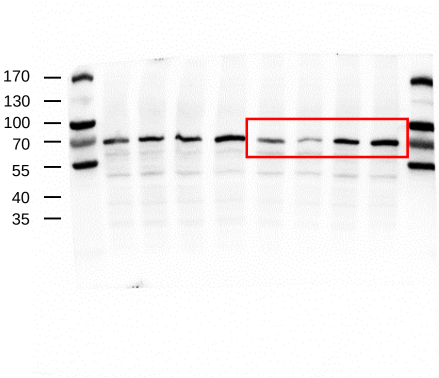

Figure 5D_p65

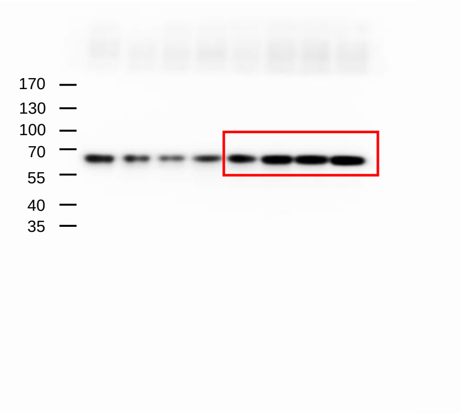

Figure 5D_β-actin

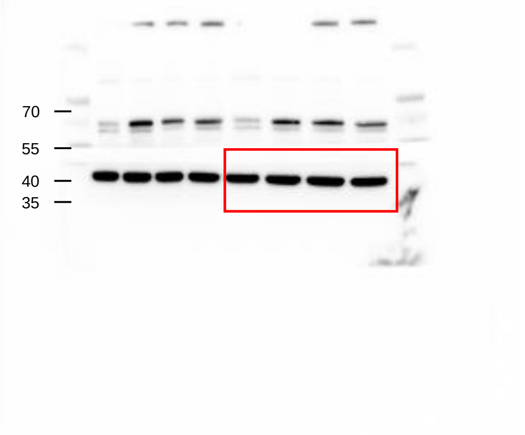

Supplementary Figure 9

Supplementary Figure 2C_pJNK

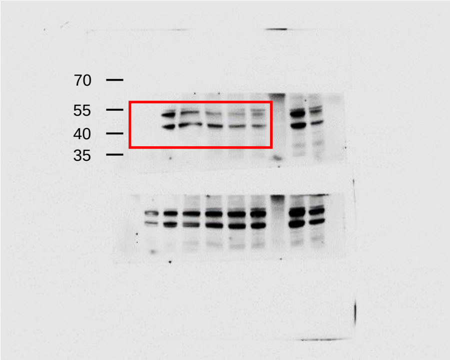

Supplementary Figure 2C_JNK

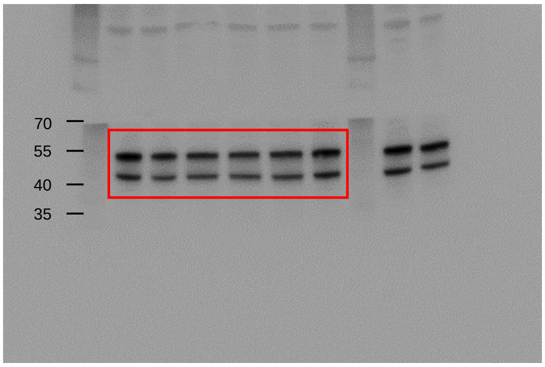

Supplementary Figure 2D_ pNFκB p65

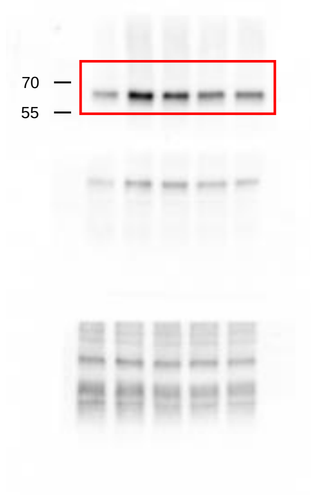

Supplementary Figure 2D_ p65

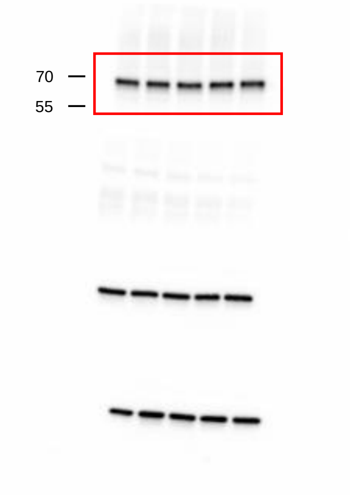

Supplementary Figure 2D_ pJNK (Long expose)

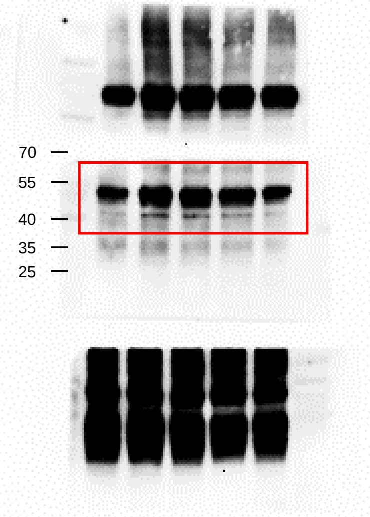

Supplementary Figure 2D_ pJNK (Short expose)

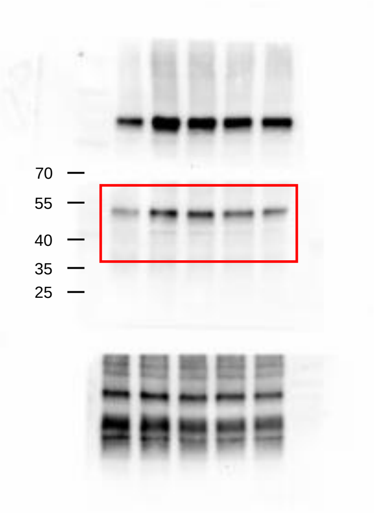

Supplementary Figure 2D_ JNK

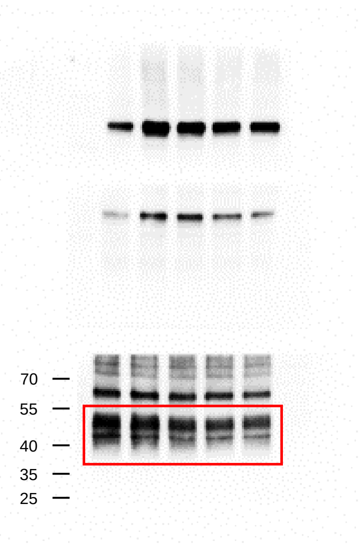

Supplementary Figure 2D_ GAPDH

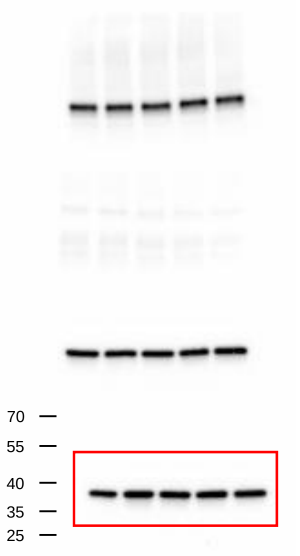

Supplementary Table 1

A

Primers	Forward Sequence (5'-3')	Reverse Sequence (5'-3')
hIL8	TTTCAGAGACAGCAGAGCACACAAGC	CCTTCACACAGAGCTGCAGAAATCAG
hFGFR1	GGCATGGAGTATCTGGCCTC	CCATCCACTTCACAGGCAGT
hEXT1	CGTGCCTCTTTGTCCTGAGT	ATGTCAAACCCCACGTCCTC
hGAPDH	GAAGGTGAAGGTCGGAGTC	GAAGATGGTGATGGGATTTCC

B

Antibody	Host	Company	Catalog	Notes
IκBα	Rabbit	Cell Signaling	#9242	WB (1:500)
p65	Rabbit	Cell Signaling	#8242	WB (1:500)
pp65 (Ser536)	Rabbit	Cell Signaling	#3033	WB (1:500)
JNK	Rabbit	Cell Signaling	#9242	WB (1:500)
pJNK	Rabbit	Cell Signaling	#4668	WB (1:500)
β-actin	Mouse	Signa Aldrich	A5441	WB (1:5000)
GAPDH	Mouse	Abcam	ab8245	WB (1:5000)
α-SMA	Mouse	Dako	M0851	IF (1:75)
CD31	Rabbit	Abcam	ab28364	IF (1:50)
HNA	Mouse	Abcam	ab191181	IF (1:200)
Laminin	Rabbit	Sigma Aldrich	L9393	IF (1:500)

Supplementary figure legends

Supplementary figure 1. Formation of FECS-Ad and angiogenic factor expression in various stimuli during 3D formation.

(A) Live cell images of FECS-Ad formation in a well of 384 well coated with MBP-FGF2 for 24 h. (B) EVs characterization by NTA secreted from monolayer hASCs and FECS-Ad. n=5 per group. (C-D) hASCs were cultured on MBP-FGF2 surface, under hypoxia, or using hanging drop method for 24 h. Conditioned medium were prepared followed by VEGF or HGF ELISA. Effect of hypoxic condition on (C) VEGF and (D) HGF protein. ns, not significant, ***$p<0.001$, ****$p<0.0001$ (one-way ANOVA), n=3 per group. All data are presented as mean ± SD.

Supplementary figure 2. Evaluation of FGFR1 knockdown and JNK activation on MBP-FGF2 surface.

hASCs were plated on NTCP or MBP-FGF2 surface and treated with 10 nM of FGFR1 or control siRNA for 24 h. Cells were prepared, and the knockdown efficiency of FGFR1 on (A) NTCP or (B) MBP-FGF2 surface was determined by RT-qPCR. Values were normalized to GAPDH. ns, not significant, ***$p<0.001$, ****$p<0.0001$ (one-way ANOVA), n=3 per group. (C) hASCs were cultured on MBP-FGF2 surface for up to 8 h, and prepared followed by Western blot. Effects of MBP-FGF2 surface on the total and phosphorylated form of JNK. (D) hASCs were cultured on MBP-FGF2 surface for up to 24 h to form FECS-Ad. Time dependent JNK and NF-κB activation during 3D formation. All data are presented as mean ± SD.

Supplementary figure 3. Roles of EXT1 on the MBP-FGF2-mediated IL-8 expression.

hASCs were treated with EXT siRNA, and cultured on NTCP or MBP-FGF2 surface for 24 h. Total RNA and conditioned medium were prepared and analyzed by RT-qPCR and ELISA, respectively. Knockdown efficiency of EXT1 on (A) NTCP or MBP-FGF2 surface. Values were normalized to GAPDH. ns, not significant, ****$p<0.0001$ (one-way ANOVA), n=3 per group. (B) Effect of EXT1 knockdown on IL-8 RNA. Values were normalized to GAPDH. ns,

not significant, *p<0.05, ****p<0.0001 (one-way ANOVA), n=3 per group. (C) Effect of EXT1 knockdown on IL-8 protein. ns, not significant, (one-way ANOVA), n=3 per group. All data are presented as mean ± SD.

Supplementary figure 4. Effects of the concentrations of various FGF2 ligands on IL-8 expression.

(A-B) hASCs were cultured on MBP-FGF2 surface with various coating concentrations for 4 and 24 h. Cells and conditioned media were prepared and analyzed by ELISA. (A) Effects on IL-8 expression in cells. ns, not significant (one-way ANOVA), n=6 per group. (B) Effects on IL-8 secretion. ns, not significant (one-way ANOVA), n=6 per group. (C-D) hASCs were plated and treated with various concentrations of soluble form of MBP-FGF2 or FGF2 for 4 and 24 h. Conditioned media were harvested and analyzed by ELISA. (C) Effects of soluble MBP-FGF2 concentration on IL-8 protein. ns, not significant, ****p<0.0001 (one-way ANOVA), n=3 per group. (D) Effects of soluble FGF2 concentration on IL-8 protein. ns, not significant, ****p<0.0001 (one-way ANOVA), n=3 per group. All data are presented as mean ± SD.

Supplementary figure 5. Effect of hASC or FECS-Ad co-culture on HUVEC tube formation.

GFP-HUVECs plated on matrigel were co-cultured with hASCs or FECS-Ad in transwell plate for 16 h. (A) Effect of hASC or FECS-Ad co-culture on the tube formation of HUVECs. (B) Effect on the total tube length of HUVECs. **p<0.01, ****p<0.0001 (one-way ANOVA), n=5 per group. (C) Effect on the number of branching points of HUVECs. **p<0.01, ****p<0.0001 (one-way ANOVA), n=5 per group. (D) Effect of FECS-Ad co-culture number on HUVEC tube formation. Formation of tubular structure was measured by (E) the total length of the tube and (F) the number of branching points. ****p<0.0001 (one-way ANOVA), n=5 per group. All data are presented as mean ± SD.

Supplementary figure 6. Histological analysis of ischemic hindlimb.

One day after HLI induction, mice were i.m. injected with PBS, FECS-Ad, or IL-8 silenced

FECS-Ad. Thigh and TA muscles were isolated and immunostained with α-SMA. Nuclei were stained with DAPI. α-SMA staining of (A) ischemic thigh and (B) TA of each group at 1, 7, 14, and 28 days post-surgery. n=4 per group. Scale bar, 40 μm. Percentage of α-SMA-positive cells in (C) ischemic thigh and (D) TA. Normal group was presented as 100%. ns, not significant between PBS and FECS-Ad group, *, $p<0.0001$ compared to PBS group, #, $p<0.0001$ compared to FECS-Ad (IL-8 KD) group (two-way ANOVA). n=4 per group. (E) HNA staining of ischemic thigh of PBS and FECS-Ad group at 3 days after surgery. Scale bar, 20 μm. (F-G) Thigh and TA muscles were isolated and followed by H&E staining. Histological analysis of (F) ischemic thigh and (G) TA of each group at 7 and 28 days after surgery. Scale bar, 50 μm. All data are presented as mean ± SEM.

Supplementary figure 7. The unprocessed western blot images for Figure 4.

Supplementary figure 8. The unprocessed western blot images for Figure 5.

Supplementary figure 9. The unprocessed western blot images for Supplementary figure 2.

Supplementary video 1.

Real-time video of FECS-Ad formation in a well of 384-well microplate coated with MBP-FGF2.

Supplementary table 1. Primers and antibodies used in this study

(A) Nucleotide sequences of primer used for RT-qPCR analysis. (B) Antibodies used for Western blot hybridization and immunofluorescence.
